# Encapsulation of Flavours and Fragrances into Polymeric Capsules and Cyclodextrins Inclusion Complexes: An Update

**DOI:** 10.3390/molecules25245878

**Published:** 2020-12-11

**Authors:** Diego Romano Perinelli, Giovanni Filippo Palmieri, Marco Cespi, Giulia Bonacucina

**Affiliations:** School of Pharmacy, University of Camerino, Via Gentile III da Varano, 62032 Camerino, Italy; gianfilippo.palmieri@unicam.it (G.F.P.); marco.cespi@unicam.it (M.C.); giulia.bonacucina@unicam.it (G.B.)

**Keywords:** encapsulation method, essential oil, polymeric capsules, coacervation, inclusion complex, volatiles, nanofibers, aromas, electrospinning

## Abstract

Flavours and fragrances are volatile compounds of large interest for different applications. Due to their high tendency of evaporation and, in most cases, poor chemical stability, these compounds need to be encapsulated for handling and industrial processing. Encapsulation, indeed, resulted in being effective at overcoming the main concerns related to volatile compound manipulation, and several industrial products contain flavours and fragrances in an encapsulated form for the final usage of customers. Although several organic or inorganic materials have been investigated for the production of coated micro- or nanosystems intended for the encapsulation of fragrances and flavours, polymeric coating, leading to the formation of micro- or nanocapsules with a core-shell architecture, as well as a molecular inclusion complexation with cyclodextrins, are still the most used. The present review aims to summarise the recent literature about the encapsulation of fragrances and flavours into polymeric micro- or nanocapsules or inclusion complexes with cyclodextrins, with a focus on methods for micro/nanoencapsulation and applications in the different technological fields, including the textile, cosmetic, food and paper industries.

## 1. Introduction

Flavours and fragrances are a large class of compounds widely employed as additives in different technological fields, including food, cosmetics, textiles and others, mainly to ameliorate the olfactory and gustatory sensations of the product [1,2]. They comprise both synthetic and naturally occurring molecules, such as essential oils (EO) and aroma compounds [3,4]. Especially those of natural origin, which are mostly derived from plants, possess, in addition to sensory properties, also various biological activities (e.g., antibacterial, antiviral, antifungal, antiprotozoal, insect-repellent, anticancer, antidiabetic, anti-inflammatory and antioxidant) that raise the interest around this class of compounds [5,6]. Besides the large potential of exploitation, the major drawbacks regarding their use are related to the volatility and chemical instability [7]. Indeed, most of these compounds are sensitive to light, heat or oxygen; therefore, they can be deteriorated during the manufacturing process and reduce or lose their shelf-life activity during storage and consumer manipulation [8]. 

To overcome these concerns, different encapsulation strategies have been applied, aiming to prevent the evaporation of volatile compounds and protect them from degradation [9]. Through encapsulation, the compounds are protected by a shell of a different nature (e.g., polymeric, inorganic, lipid or mixed), which acts as a diffusion barrier, thereby enhancing their retention, controlling the release and prolonging the chemical stability [10]. Encapsulation can be achieved using several techniques depending on the nature of the wall material and the fragrance itself, leading to the formation of micro/nano cargoes such as capsules, spheres or vesicles. Both the encapsulation of flavours and fragrances in cargoes of nanometric (nanoencapsulation) and of micrometric (microencapsulation) size have been widely investigated. However, microencapsulation has some advantages over nanoencapsulation, such as a higher payload, better control on the release and an easier processing and industrial scalability [11]. Although several materials of a different nature have been proposed as shells for the encapsulation of fragrances and flavours [12,13,14], polymers and cyclodextrins (CDs) still remain the most employed in all technological fields [15]. Particularly, polymers both of natural or synthetic origin have been reported to successfully encapsulate flavour and fragrances into single or multi-layered core-shell micro- or nanocapsules [16,17,18]. These capsules resulted in being highly versatile for the encapsulation of volatile compounds, thanks to the large variety of polymers and methodologies available (e.g., coacervation and interfacial polymerisation), through which their chemical–physical properties can be tuned [19,20]. Therefore, polymeric capsules can provide an easy handling and processing of this class of chemical compounds, guaranteeing, at the same time, a satisfactory protection from evaporation or degradation, good mechanical properties and the possibility of modulating or controlling the release at different conditions [21]. Besides, molecular inclusion complexation with CDs has been also widely exploited [22]. CDs represent a simple and relatively affordable material, resulting effectively in the encapsulation of aroma and volatile compounds [23]. CDs are a family of cyclic oligosaccharides (α-CD, β-CD and γ-CD), composed of six, seven or eight glucosyl units, having a hydrophilic outer surface and a hollow hydrophobic cavity, able to host lipophilic “guest” molecules with a defined size, shape and stoichiometry of interactions. They were employed for the encapsulations of a large variety of volatiles, such as EOs, plant flavours and spices, with the aim to mask unpleasant smells and tastes, to convert them into solid crystalline forms and to improve physical and/or chemical stability [23,24].

The present review addresses the recent literature, mainly focusing on papers published between 2018 and 2020 related to the encapsulation of flavours and fragrances into polymeric capsules and inclusion complexes with CDs. Particular attention was devoted to the applications of these encapsulated systems in different technological fields, such as textiles, cosmetics, food and paper industries.

## 2. Methods of Preparation for Micro/Nanoencapsulation of Flavours/Fragrances in Polymeric Capsules and Molecular Inclusion Complexes

### 2.1. Polymeric Capsules

Different methods have been reported in the literature for the encapsulation of flavours and fragrances in polymeric capsules [16]. The choice of the most suitable technique depends on the different types of core and shell materials, on the properties that the final micro- and nanosystems have to possess in terms of size, shell thickness and permeability and on the desired release rate of the active molecule. In addition, the final application of the capsules can also affect the selection of the more suitable encapsulation process, which could be tailored as a function of the intended use. Generally, these techniques can be divided into three major categories—specifically, chemical methods (e.g., in situ polymerisation, emulsion polymerisation and interfacial polymerisation); physical-chemical methods (e.g., emulsification and coacervation) and physical-mechanical methods (e.g., spray drying, freeze-drying, electrodynamic methods and extrusion) [9,25]. These methods have been extensively reviewed in the last years, highlighting their strengths and weaknesses, in relation also to the different applications for which they have been applied. An overview about the recent advances in the applied methods of micro/nanoencapsulation for fragrances and flavours in polymeric capsules and the formation of molecular inclusion complexes with CDs is presented here.

Although chemical methods for encapsulation are suitable for capsules formed by shells made of synthetic polymers, the so-defined physical-chemical and physical-mechanical methods can be employed both for natural and synthetic polymers. However, chemical methods are generally preferred for synthetic polymers, since, in most cases, they are more effective in controlling the size, the shape of the capsules and assuring a high loading capacity and encapsulation efficiency [16,26]. These methods include in situ polymerisation, emulsion polymerisation and interfacial polymerisation [27,28,29]. Recently, a new approach has been proposed based on the free-radical crosslinking copolymerisation of a double oil-in-water-in-oil (O/W/O) emulsion to prepare synthetic polymeric capsules encapsulating fragrances. This strategy has the advantage of separating the polymerisation process, occurring in the aqueous phase that contains monomers, crosslinkers and an initiator, from the fragrance compartment. In this way, possible undesired reactions involving the fragrance during the polymerisation process are avoided [30]. 

Coacervation is a largely employed method since the 1950s for the micro- and nanoencapsulation of different compounds based on the physicochemical process of phase separation in which a polymeric dispersion can form a liquid polymer-rich phase, known as coacervate, at specific conditions [9]. Coacervation can be classified as simple or complex. In simple coacervation, the polymer is salted out by the action of electrolytes, or desolvated by the addition of a water miscible nonsolvent, while complex coacervation is essentially driven by the attractive forces of oppositely charged polymers. The encapsulation process can be performed in an aqueous phase for the encapsulation of hydrophobic water insoluble materials or in the organic phase or via a preliminary double-emulsification step for the encapsulation of hydrophilic compounds [31,32]. Therefore, coacervation allows the encapsulation of different kinds of functional ingredients (solid or liquid core materials), including flavours and fragrances, to be utilised in many industrial sectors, such as food, cosmetics or pharmaceuticals [33,34]. The complex coacervation process has been largely exploited to obtained polymeric capsules containing fragrances, flavours and EOs in the core and biopolymers such as proteins (e.g., gelatin and silk fibroin) and polysaccharides (gum arabic, gum tragacanth, pectin, chitosan, agar, alginate, carrageenan and sodium carboxymethyl cellulose) as shell materials [35,36]. Recently, polyelectrolyte complexes using cationised casein, as an alternative polycation, and sodium alginate were prepared via complex coacervation without crosslinking agents. These complexes were stable and suitable for a controlled release of vanillin fragrance [37]. In another recent study, oregano EO was encapsulated through complex coacervation using gelatin and chia mucilage as an alternative to plant-derived gums. The obtained nanocapsules were compared to those prepared with the standard polyelectrolyte combination gelatin/arabic gum after a spray-drying process. A high EO entrapment both before and after spray-drying was achieved using the combination gelatin/chia mucilage. Moreover, the particle size after drying was actually lower than the control formulations, suggesting the potential use of a gelatin/mucilage combination for the encapsulation of EOs in different applications [38].

Phase separation of a polymer from a colloidal dispersion can be also achieved using a vapour phase as the antisolvent, the so-called vapour-induced phase separation (VIPS). This technique has been widely employed for the preparation of films, membranes and hydrogels, but it has been recently proposed for the preparation of microcapsules. A complex mix of fragrances have been encapsulated in cellulose acetate microcapsules via the VIPS technique. The obtained capsules had a core-shell architecture, high encapsulation capacity and stability up to one year at room temperature, showing no fragrance diffusion without external stimuli at a dry state [39].

Among physico-mechanical methods, the currently most employed for the encapsulation of flavours and fragrances is still spray-drying. It has been reported for flavour encapsulation that around 80–90% of the encapsulated products are obtained by spray-drying; then, by spray-chilling (5–10%), melt extrusion (2% to 3%) and melt injection (∼2%) [40].

Specifically, spray-drying is one of the most common methods used for several reasons, such as equipment availability and simplicity, the possibility to use a wide variety of encapsulating agents, large-scale production, good efficiency and reduced processing costs [41,42]. On the other side, a relevant loss of aroma compounds could occur during the spray-drying process due to the eventual chemical reactions activated at the operating temperature among the flavour and fragrance constituents or volatile diffusions through the shell and the consequent evaporation into the environment [43].

Spray-drying has been extensively employed for the microencapsulation of EOs, using several wall materials, especially polysaccharides (e.g., chitosan and carrageenan) or gums [44,45]. Specifically, the ingredient to be encapsulated is added to the carrier (the ratio of core-to-carrier can be optimised for each individual combination), and then, the dispersion is fed into the spray-drying chamber, passing through an atomiser (e.g., spray nozzle). The atomisation occurs thanks to the circulating hot air that allows the evaporation of the aqueous medium. The dispersed carrier materials should be soluble in water and have low viscosity at high concentrations to assure efficient drying properties [41,46]. The factors influencing the spray-drying process, as well as the characteristics of the obtained EO-loaded capsules, have been investigated. In a study, the impact of the wall composition (whey protein isolate, maltodextrin and sodium alginate) has been evaluated in terms of the formation and stability of cinnamon EO microcapsules produced by spray-drying [47]. In another one, the effect of using a reduced pressure and an oxygen-free environment during the spray-drying process (vacuum spray-drying, VSD technique) was examined in comparison to the conventional spray-dryer (SD technique) for the encapsulation of orange EO using maltodextrin and octenyl succinic anhydride-modified starch as the wall material. The VSD technique provides microcapsules with a smaller size and higher encapsulation efficiency than those from the standard technique [48].

Spray-chilling, also known as spray-cooling, spray-congealing or prilling, is another congener technique utilised for the microencapsulation of flavour compounds, especially when lipids are employed as wall materials [49,50]. Spray-chilling is similar to spray-drying, but a cooling chamber instead of a drying chamber is required. This technique is also easy to use and to scale up with a lower loss of flavours by diffusion, thereby avoiding organic solvents and the application of a high inlet air temperature [51]. One disadvantage is the poor control of the particle size and moderate yields.

Electrohydrodynamic processes such as electrospinning and electro-spraying can be also used for the encapsulation of flavours and fragrances [52], allowing, generally, the production of micro- or nanofibers from a polymeric dispersion using a spinneret by applying a high voltage potential or particles in the nozzle through liquid atomisation by electric forces [53]. Indeed, these techniques use different concentrations of the polymeric dispersion that give rise to nanofibers by electrospinning with high concentrations of the polymer or fine droplets/particles when a low polymer concentration is used in the electro-spraying [54]. Since these methodologies do not require heating treatments, they are very promising for the encapsulation of heat-sensitive compounds such as flavours, fragrances and EOs [55,56,57]. Different polymers have been evaluated for the formation of nanofibers encapsulating volatile compounds, such as cellulose derivatives [58], biodegradable polyesters [59], dendrimers [60] or polysaccharides such as seed gums and mucilages [61,62]. In the last years, new advances in the field of electrospinning-based techniques have been introduced as coaxial electrospinning/spraying and emulsion electrospinning/spraying, enabling the production of core-shell fibres and particles [63,64,65]. An example is from the work of Dehcheshmeh and Fathi, in which an aqueous saffron extract was encapsulated in core-shell nanofibers via the coaxial electrospinning technique. The shell was formed by zein, while the core was made by gum tragacanth, in which the saffron extract was dispersed. The results of this research showed that produced core-shell nanofibers were thermostable, assuring the stability and a satisfactory entrapment for the saffron extract compounds, which were slowly released in saliva, hot water, a gastric simulant and intestinal simulant media [66]. Core/shell nanofibers containing cinnamon oil were also successfully obtained by the emulsion electrospinning technique, using poly(vinyl alcohol) as the water phase. These nanofibers contained up to 20% *w*/*w* of cinnamon oil and showed a continuous release of the major volatile components (cinnamaldehyde, eugenol and caryophyllene) for up to 28 days [67]. Recently, another electrospinning technique has been proposed, i.d. needleless electrospinning, more suitable for the production of large-scale batches, since no needles are used, thereby avoiding clogging limitations. Differently from the more common technique, in which the fibres form due to the mechanical forces and geometric characteristics of the needle, it is based on the self-formation of the electro-spun-induced fibres on an open-surface electrode [68]. Needleless electrospinning has been employed for the nanoencapsulation of cinnamic aldehyde in zein nanofibers [69] or the nanoencapsulation of thyme EO in chitosan/gelatin nanofibers [70]. The obtained nanofibers showed bactericidal effects and, after mixing in sausage batter, do not alter the colour, texture and sensory characteristics of the final food product.

Melt extrusion is another “traditional technique” employed in the past decades for the encapsulation of flavours and fragrances [71]. It consists of the melting of the polymer with the plasticiser and the subsequent mixing of the compound to be encapsulated. The obtained melt is forced out of the extruder orifice under high pressure. Droplets originate from the action of the surface tension, gravitational or frictional forces, which result in the formation of solid particles when quickly dried. A variant is represented by the co-extrusion method, enabling the formation of core-shell particles. Specifically, the liquid active ingredient and the solubilised wall material are pumped, separately in two streams, through a concentric nozzle. Droplets are formed by applying vibration on a laminar jet, giving particles after drying. These techniques require “mild” operating conditions, and they have been employed for carbohydrates, dextrins or starch-based polymers [72]. In a recent work, different blends of a modified-starch (i.d. octenyl succinate starch) and malto-polymers with different molecular weights were investigated to optimise the microencapsulation of orange oil through the twin-screw extrusion process. The study highlighted how the matrix composition, the amount of water in the mixture and the degree of starch gelatinisation affected the oil payload [73].

Over the years, alternative novel methods have been investigated for the encapsulation of fragrances and flavours. Indeed, supercritical CO_2_ (sCO_2_) technologies have been employed to formulate particles or capsules with a wide variety of polymeric materials [74]. In these processes, supercritical CO_2_ can act either as a solvent, solute or antisolvent, giving rise to different techniques (e.g., Rapid Expansion of Supercritical Solutions, RESS and supercritical antisolvent, SAS techniques). CO_2_ methodologies are versatile and scalable, allowing a formulation process in a completely anhydrous medium, by obtaining noncontaminated products, high encapsulation efficiencies, customised particle properties and good scalability. The characteristics of the particles/capsules can be tuned by employing supercritical CO_2_ at different operating conditions (e.g., temperature, pressure) [75]. Recently, the particle from a gas saturated solution (PGSS) technique, using sCO_2_ as the solvent at a moderate pressure and temperature, was employed for the encapsulation of eucalyptol in polyethyleglycol/polycaprolactone microparticles and of *Citrus aurantifolia* EO in polyethylene glycol/lauric acid microparticles, demonstrating satisfactory entrapment efficiency and controlled release [76,77]. sCO2-based technique alternatives to the conventional ones can be also employed for encapsulation, such as the supercritical fluid extraction of emulsions (SFEE). This technique is based on the removal of the organic phase solvent in fractions of seconds as the time scale by sCO2, leading to the rapid precipitation of compounds dissolved in it. In general, very small and highly homogeneous particles are obtained. Lima Reis et al. reported for the first time the encapsulation of an EO (i.d. *Laurus nobilis* EO) using the SFEE technique. A chemically modified food starch was used as the encapsulating agent. The efficiency of encapsulation by the SFEE process was found to be favoured by the increase in EO concentration and the final dried particles demonstrated to be effective in the protection of this highly volatile compound [78].

**Figure 1:**
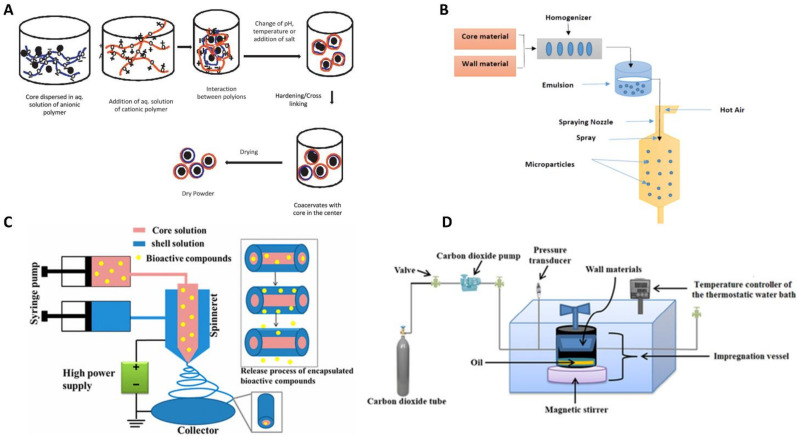
Schematic representation of the micro/nanoencapsulation process by (**A**) complex coacervation, (**B**) spray-drying, (**C**) coaxial electrospinning and (**D**) supercritical fluid technology (image adapted from [31,34,45,54]).

Figure 1 provides a schematic representation of some micro/nanoencapsulation processes (complex coacervation, spray-drying, coaxial electrospinning and supercritical fluid technology) successfully employed for the encapsulation of fragrances and flavours.

### 2.2. Molecular Inclusion Complexes with CDs

The formation of molecular inclusion complexes using CDs is a very common microencapsulation approach, widely investigated for different purposes [79,80,81,82]. It is based on the stoichiometric hydrophobic interactions via dynamic equilibrium between CDs and the complexed substance, which is entrapped in the hydrophobic cavity of CDs [83]. The formed host–guest complexes demonstrated being effective to improve the stability and prolonged release of large amounts of fragrances, flavours, EOs and volatiles [84]. Apart from the production of continuous and extensive studies aimed to study the interactions and, therefore, the encapsulation capability of CDs towards volatile compounds [85,86,87], a flourishing literature has been recently produced regarding the processing of molecular inclusion complexes by electrospinning to obtain micro- and nanofibers in which fragrances and volatile compounds are incorporated [88,89].

In the last years, the same research group has published several works reporting the encapsulation of molecular inclusion complexes of volatile compounds incorporated in polymeric fibres, mats or webs by electrospinning. A polymer-free electrospinning approach was applied on CD inclusion complexes to enhance the water solubility; improve the high temperature stability and control the release of carvacrol [90], tymol [91], camphor [92], menthol [93], limonene [94], citral [95], cineole and p-cymene [96] and eugenol [97]. In other studies, the volatiles/inclusion complexes with CDs were incorporated in a biopolymer matrix as zein [98,99], o pullulan [100], semisynthetic polymers such as cellulose acetate [101] or synthetic polymers such as poly(3-hydroxybutyrate-co-3-hydroxyvalerate) (PHBV) [102] via electrospinning. These polymers have been used for the formation of edible or biodegradable antimicrobial films, as well as porous membranes for packaging or biomedical applications.

## 3. Applications of Micro-/Nanoencapsulated Fragrances and Flavours

Micro- and nanocapsules/spheres, as well as molecular inclusion complexes with CDs, have been largely employed as protective carriers for aroma compounds (fragrances, aromas and flavours) in different technological fields [25,103]. The following paragraphs summarise the main experimental studies recently conducted on the design and application of micro- and nanocapsules/spheres in the textile, food, cosmetic and paper production fields. 

### 3.1. Textile Applications

**Figure 2:**
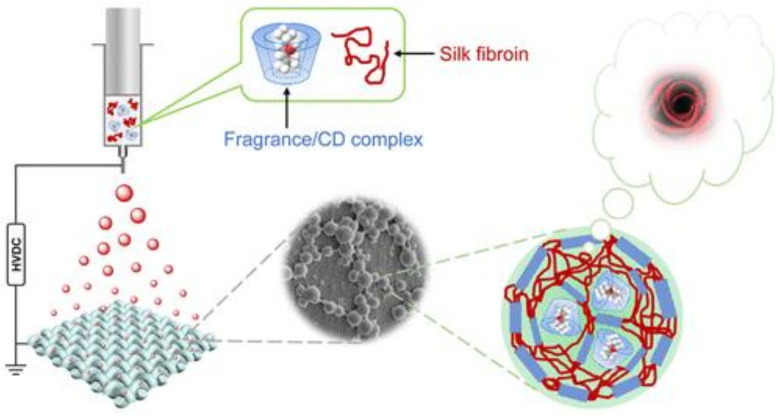
Direct deposition of fragrance-loaded nanoparticles onto fabric surfaces by electro-spraying (reproduced from [113]). CD: cyclodextrins.

Textiles represent one of the most investigated applications for micro- and nanospheres/capsules encapsulating fragrances and aromas. These encapsulated volatile compounds have been employed for several years in textile-finishing processes, such as fabric conditioners to impart freshness and odour control [104,105,106,107]. Through encapsulation, fragrances are retained and released for a long time [108]. Moreover, the sensation of the added encapsulated fragrances can be preserved also after several washing-drying cycles (up to 25); therefore, the attractiveness of the product to the consumers is improved [109,110]. Encapsulated perfumes and EOs have been added in scarves, ties, lingerie and other garments, as well for home textiles, such as sofa coverings, curtains and cushions for aromatherapy [106,111]. Perfumes and aromas can be directly applied on textiles; however, their scarce affinity to fabric fibres and their chemical volatility limit their permanence. Thus, encapsulation promotes a prolonged duration of aroma sensations due to the controlled release of the fragrance. For this purpose, several types of fabrics can be processed with encapsulated fragrances and aromas, such as cotton, silk and synthetic fibres (polyamide or polyester). These micro- and nanocapsules/spheres can be added to textiles using different techniques, such as impregnation, spraying, coating or stamping [103,112]. The encapsulation of fragrances and aromas is still achieved through traditional methods such as simple or complex coacervation, as well as the inclusion encapsulation method or interfacial polymerisation. However, other “innovative” encapsulation processes for fragrances and aromas have been recently explored in textile applications. Ye et al. proposed an electro-spraying method using aqueous media to prepare composite nanospheres made up of silk fibroin and β-CD encapsulating rose oxide or D-limonene (Figure 2). The nanospheres have an aroma encapsulation higher than 90% and were deposited directly on silk fabric. The fragrances were released with zero-order kinetics, guaranteeing a low rate and constant release profile. Noticeably, the composite nanospheres were retained at a higher percentage (more than 80%) after 10 runs of washing with water, demonstrating its applicability in the textile field [113].

The retention of fragrances and aromas, especially after washing or rubbing, depends on the penetration of microcapsules and nanocapsules into the spacing of textiles during the finishing process. To address this, in a work, a series of micro-/nanocapsules, with a size suitable for the pore spacing of cotton textiles and formed by citronella oil as the core material and chitosan as the wall material, was prepared through a microemulsion approach. These micro-/nanocapsules were applied on the textile through vacuum impregnation. The matching between the spacing of the pore sizes of cotton textiles and the sizes of micro-/nanocapsules was assessed via the retention of aromatic compounds in the finished cotton textiles after several washing cycles (washing durability). Indeed, the aromatic retention of cotton textiles finished by nanocapsules was much greater than the same textiles finished with microcapsules (28.84% vs. 1.55%) after 10 cycles of washing. The authors demonstrated that nanocapsules can penetrate better into the pores of the cotton textiles [114]. To overcome the issue related to the poor combination fastness and duration in the textiles, several approaches were employed in the past, using chemical binders or crosslinking agents. Recently, Ma et al. exploited electrostatic adsorption and immobilisation to retain nanocapsules loaded with lavender essence on cotton textiles. Firstly, the textile was positively charged through quaternary ammonium cationisation to promote the adsorption of nanocapsules with a negatively charged surface. The in-situ immobilisation was achieved via the diffusion and permeation of an alkali solution, leading to a chemical bond between nanocapsules and the textile fibres at the position of absorption. The encapsulated fragrance was released continuously for 120 days, and 91.19% of the essence still remained entrapped in the textile after five washing cycles. The authors proposed this method as a simple and “green” approach for the preparation of nanocomposite textile materials for different applications. 

On the other side, the encapsulation of fragrances and aromas was pursued recently for the fabrication of “smart textiles” with additional functional properties [115], such as antibacterial, UV protection, moisturising and skin treatments, body temperature regulation and insect repellence, depending on the action of the encapsulated fragrances, aromas or EOs [116,117,118,119]. An example of encapsulation for UV protection in textiles is from the work of Chen et al., in which the one-step fabrication of cellulose/silica hybrid microcapsules via an emulsion solvent diffusion method was reported [120]. These microcapsules were loaded with lavender fragrance oil and embedded into a polysiloxane coating. This coating ensured a controlled release of the EO and an excellent UV protective property, even after 30 repeated abrading/heating cycles, thanks to the grafting onto the particle shell of UV absorbers. The authors proposed the use of this material for sports clothing, curtains and other outdoor textiles [121].

Among the different classes of functional textiles, those with the most potential exploitation are the cosmetic textiles or cosmetotextiles. They are defined from the European Cosmetic Directive (76/768/EEC) Article l as “any textile product containing a substance or preparation that is released over time on different superficial parts of the human body, notably on human skin, and containing special functionalities such as cleansing, perfuming, changing appearance, protection, keeping in good condition or the correction of body odours” [122]. In these textiles, cosmetic ingredients are adsorbed or incorporated inside the cotton fibres of clothes and garments to be transferred after contact to the skin at a dose enough to impart some cosmetic benefits [123].

The active ingredients, including fragrances and aromas, are generally encapsulated and released from the fabric to the skin upon the action of different triggering events, such as changes in the pH or temperature, sweating and rubbing [124]. As for the other functional textiles, the encapsulation of the active ingredients allows for a prolonged release, even after a few washing–drying cycles [125]. The washing durability is enhanced when the active ingredient is incorporated inside the fabric fibres with respect to the application by coating or impregnation. The encapsulated active ingredient embedded or adsorbed onto a cosmetotextile can exert any cosmetic action, including skincare, antiaging or odour control. Encapsulated aromas and fragrances have been incorporated in cosmetotextiles for perfuming or deodorising purposes, thereby controlling odours resultant from daily activities and physical exercise. In a recent work, two strategies were reported for the release of β-citronellol from cotton functionalised with Carbohydrate-Binding Module (CBM) proteins. The first strategy was based on the odorant-binding proteins (OBPs) as a container for the fragrance, while the second one exploited the high cargo capacity for β-citronellol of liposomes. These two carriers were bound to the cotton fabric via CBM proteins. These two approaches were able to differentiate and control the release of β-citronellol after exposure with an acid sweat solution. Indeed, the release was faster for the OBP-based approach with respect to the immobilised liposomes on the functionalised cotton (31.9% vs. 5.9% of the initial amount after 90 min, respectively). Therefore, the first strategy result is more suitable for applications in which a high amount of fragrances should be released in a shorter time, while the second strategy is potentially employed for fabrics from which the fragrance should desirably be released in a prolonged and controlled manner [126]. The most used coolant agent, menthol, which is able to penetrate through the stratum corneum, reaching the nerve termination and providing a freshening sensation, was loaded in a core-shell nanocapsule impregnated within nonwoven fabric. The nanocapsules assured a rapid penetration of menthol inside the deeper skin layers, preferentially through hair follicles and trans-epidermal absorption routes [127]. Similarly, citronella oil was encapsulated in acacia gum microcapsules, which were dripped onto a nonwoven fabric. Microencapsulation by spray-drying reduced the volatility, with a prolonged release up to 16 weeks, and decreased the irritation potential of nonencapsulated citronella oil, as evaluated by the nonanimal hen’s egg test-chorioallantoic membrane (HET-CAM) assay [128].

Table 1 and Table 2 summarise the recent studies reporting the microencapsulation and nanoencapsulation of fragrances and flavours for textile applications, respectively.

### 3.2. Food Applications

Another application in which the micro- and nanoencapsulation of fragrances and flavours research has been focused on is related to food [143]. As for the other active ingredients, the encapsulation of fragrances and flavours has been exploited in food processing and for the design of active food packaging [40]. In the food industry, encapsulated flavours and fragrances have been widely used to ameliorate taste and/or odour, to adjust the nutritional value and to prolong the shelf-life of food [144,145,146]. As such, food quality has improved, with positive implication in terms of consumer satisfaction and food consumption [147]. For instance, fragrances and flavours are volatile compounds and are prone to evaporation during several food-processing operations or storage of the final product. Moreover, they can undergo chemical instability due to oxidation in the presence of air and light, moisture or high temperature, leading to chemical degradation and possible interactions with other food additives [148]. In this regard, these compounds can be stabilised by encapsulation or complex formations. In addition to overcoming these concerns, encapsulation and/or complex formations improve also the manageability of these volatile food additives, guaranteeing stability and a simpler and standardised dosing. A classic example of the encapsulation of flavours in food technology is coffee aroma. Coffee aroma compounds are a mixture of pyridines, pyrazines, ketones, furans, etc. contained in the oil extracted from roasted coffee. These compounds are considered as flavouring agents to enrich the aroma, especially in instant coffee formulations. Roasted coffee oil is composed of several unsaturated fatty acids sensitive to oxidative degradation in the presence of air. Therefore, microencapsulation has been proposed as a strategy to preserve the freshly brewed coffee aroma in instant coffee products for a prolonged time after the first opening of the packaging. In addition, microencapsulation can be employed to control the release of these coffee aroma compounds over time. Specifically, roasted coffee oil was encapsulated in a modified food starch derived from waxy maize, and the resultant microcapsules were added to the formula of soluble coffee and instant cappuccino products with the aim of modulating the release of volatile organic compounds (VOC). The addition of microparticles improved the quality of the products in terms of aroma intensity, and the authors demonstrated how the composition of the product can affect the aroma release profile [149]. Among all fragrances and aromas, EOs obtained from a large botanical variety of plants are the most encapsulated substances in food [40,150]. They are used to provide a pleasant smell to food or to cover the original odour, configuring the olfactory sensation as a product identity marker. Being volatile liquids, their direct incorporation in food is not straightforward. Therefore, the food industry generally employed encapsulated or complex EOs, since these technological approaches both stabilised the components of EOs and increased their manageability. Complexation with β-CDs and encapsulation by simple or complex coacervation are still the most investigated in recent scientific studies. Different EOs have been employed for their antioxidant and antimicrobial effects, exploited for fruit preservation. Syringa EO was microencapsulated by the formation of complexes with β-CDs and used as an antifungal agent against *Botrytis cinerea* and *Alternaria alternata* to improve the quality attributes and storage behaviours of peaches [151]. Similarly, microcapsules of *Zingiber officinale* EO were prepared using chitosan and carboxymethyl cellulose as shell materials to investigate the effects on the postharvest quality and prolonged the shelf-life of jujube fruits in terms of morphologic characteristics and some parameters as soluble solid contents, titratable acidity, the Red index and decay index and sensory quality [152]. The EO extracted from the leaves of Eucalyptus and incorporated into carboxymethyl cellulose (CMC) was employed to control fungal growth causing soft rot on strawberries, configured as a valid alternative to synthetic fungicides for this preharvest treatment [153].

**Figure 3:**
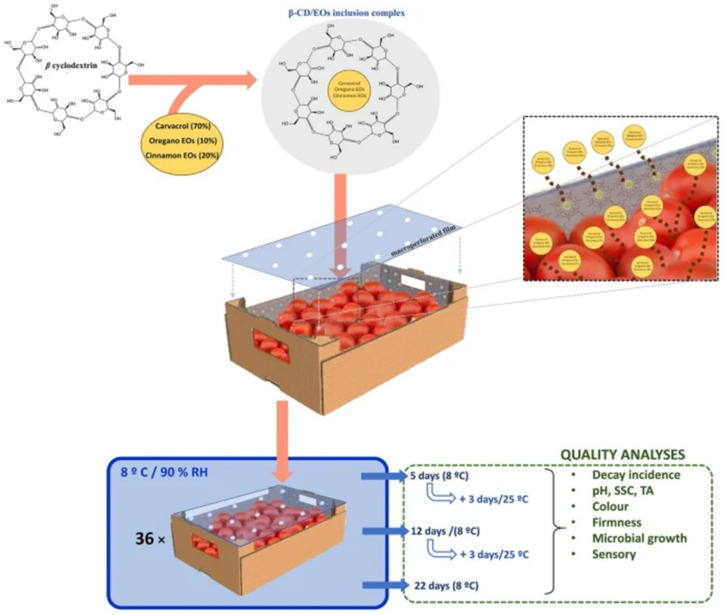
Application of essential oil-β-CD molecular inclusion complexes for the preparation of an active cardboard packaging for food storage (Reproduced from [174]).

Active packaging represents a fashionable option to preserve the quality and prolong the shelf life of food products. It refers to packaging materials, which are not inert, and does not exert only a mechanical function of enclosing food, but they “actively” interact with the atmosphere inside the packaging or directly with food products [154,155,156]. In most cases, active packaging results in being effective in preventing chemical–physical or microbiological degradation by maintaining, at the same time, the organoleptic and nutritional properties of the product (Figure 3) [157]. Studies about active packaging have increased over the years, and several EOs have been incorporated to prepare materials with antioxidant and antimicrobial properties [158]. In this field, besides the traditional encapsulating approaches such as β-CD complexation and simple or complex coacervations, nanofibers or microfibers of different compositions have been explored [159]. Cinnamon EO as an antimicrobial agent for spoilage bacteria of edible fungi was encapsulated in polyvinyl alcohol/β-CD. Then, nanofibers were formed by electrospinning and chemical crosslinking to finally obtain a film. The film was applied on the inner surface of the box containing mushrooms. The packaging based on the nanofibrous film inhibited Gram-positive and Gram-negative bacteria and prolonged the shelf life of mushrooms, especially regarding quality parameters such as hardness and colour [160]. Cinnamon EO was also encapsulated in CD nanosponges (CD-NS) as an antimicrobial agent for antimicrobial activity against foodborne pathogens, potentially employed in food packaging. CD-NS containing cinnamon EO displayed an effective antibacterial effect toward the tested bacteria. Notably, encapsulation enhanced the antibacterial activity of cinnamon EO with the respect to the nonencapsulated one, despite the slower release profile. According to the authors of the work, it represents the first study demonstrating the potential use of CD nanosponges to encapsulate and control the release of EOs in aqueous media [161]. A biocomposite for active food packaging was prepared using chitosan, β-CD citrate (β-CDcit) and an oxidised nanocellulose (ONC) biopolymer. The obtained film was then impregnated with clove EO, which was retained possibly by the formation of inclusion complexes between the components. A higher activity toward Gram-negative than Gram-positive bacteria and toward fungi than yeast was observed in comparison to chitosan film alone [162]. In another work, a saffron extract was encapsulated by electrospinning and electro-spraying techniques in zein matrices, yielding different microstructures as particles or fibres. This microstructure protected the encapsulated bioactive compounds from the saffron extract at different pH values, storage temperatures and UV light exposure, configuring these materials as potentially employed for food packaging and food healthy formulations [163]. 

Table 3 summarises the recent studies reporting the microencapsulation and nanoencapsulation of fragrances and flavours for food applications.

### 3.3. Cosmetic Applications

The European Union (EU) Cosmetics Regulation defines a cosmetic product as “any substance or mixture used for external parts of the human body (epidermis, hair system, nails, lips and external genital organs), teeth and mucous membranes of the oral cavity for cleaning them, perfuming them, changing their appearance and/or correcting body odors and/or protecting them or keeping them in good condition” [187]. In the last years, the beauty and personal care industry have become a multibillion-dollar international business with a significant growth value in emerging markets, such as Brazil, China, India, Indonesia and Argentina [188,189]. In general, there is an increasing interest in natural cosmetic formulations that generates the demand for new products reformulated by using botanical and bioactive ingredients, including fragrances and aromas, to contribute to health, beauty and wellness. Another goal to have success in such a competitive and demanding sector is the use emergent technologies, such as microencapsulation able to give innovation, functional properties and, thus, an additional value to a cosmetic product [190]. In particular, microencapsulation technologies have been proposed to increase stability, to protect against degradation and, also, to direct and control the release of active ingredients [191,192]. 

Fragrance ingredients are active ingredients commonly used in cosmetic products intended for the application to skin and hair with the purpose to release pleasant odours. In some cases, the products also labelled as “unscented” may contain fragrances to mask the unpleasant smell of other ingredients without giving a perceptible scent. The application of microencapsulation technology on the delivery of flavours and fragrances is a topic of relevant interest considering the need to improve the efficacy of a wide range of cosmetic (perfumes) and personal care (hand and body wash, toothpaste, etc.) products [192]. Fragrances are small volatile substances with scents, and their volatility is fundamental for the sensory response, despite causing an often-undesired loss during storage, limiting their effective use as additives in various products [193]. Different substances are often used to replace natural fragrances because of their poor chemical and physical stability. Among these compounds, there are, for example, synthetic nitro- and polycyclic musks used in perfumes, deodorants and detergents that are toxic and nonbiodegradable, with accumulations in the environment, aquatic organisms [194] and, also, in human milk [194]. Since natural fragrances represent a preferable alternative from a toxicological point of view, microencapsulation represents an effective strategy to overcome all the issues related to their delivery. Microencapsulation can improve the shelf life and the delivery of highly volatile fragrances, with a gradual release of the encapsulated functional ingredient. Furthermore, the encapsulation technique has a strong effect on different odour properties and consumer perceptions, such as wet odour impact, tenacity and long-lasting odour during use, that are fundamental concerns for a cosmetic product. On the other hand, the formulation of effective nano- or microcapsules needs to take into account different issues, such as the amphiphilicity of volatile compounds and the need and difficulty to obtain monodisperse microcapsules with precisely controllable shell thicknesses and shell materials. New preparation techniques have been tested to obtain microcapsules with precisely tunable sizes, highly efficient encapsulation and proper shell properties, such as a crosslinking density, polarity and thickness, to achieve the enhanced retention of fragrances [21]. Another strategy is the use of chemically functionalised biodegradable polymeric carriers able to give enhanced properties over conventional carrier materials with the advantage of being nonreactive when in contact with the human body and metabolised and removed from the body via normal metabolic pathways [33,195]. The most commonly used shell materials in cosmetics are polysaccharides (gums, starches, celluloses, CDs and chitosan) [196,197]; proteins (gelatin, casein and soy proteins) [198]; lipids (waxes, paraffin and oils) [198,199] and synthetic polymers (acrylic polymers, polyvinyl alcohol and poly(vinylpyrrolidone)) [15,200]. Inorganic materials (silicates, clays and polyphosphates) can also be used [201].

Different examples can be found in the literature on the development of systems intended for the encapsulation of fragrances with cosmetic applications. Sansukcharearnpon et al. encapsulated six fragrances: camphor, citronellal, eucalyptol, limonene, menthol and 4-tert-butylcyclohexyl acetate using the solvent displacement method and different polymer blends of ethyl cellulose, hydroxypropyl methylcellulose and poly(vinyl alcohol) as polymeric carriers. The process gave a 40% fragrance loading capacity with an 80% of the encapsulation efficiency at the fragrance:polymer weight ratio of 1:1 [202]. A more recent example was represented by the encapsulation of Kaffir lime oil, an EO from Kaffir lime leaves. It is known to possess some important bioactivities, such as antioxidant, antileukemic, antitussive, antihemorrhage, antioxidative stress and antibacterial properties, that make it a fragrance used in the food, perfumery and cosmetic industries. Nanoencapsulation were obtained through the coacervation process. Nanocapsules with uneven surface morphology and a mean size of 457.87 nm with an encapsulation efficiency of 79.07% were formulated [203]. Novel biocompatible nanocapsules (mean diameter 100  nm) based on soya lecithin and 1,2-distearoyl-sn-glycero-3-phosphoethanolamine-N-(polyethylene glycol)-2000 (DSPE-PEG (2000)) as a polymeric shell and PLGA as a core material encapsulated a lily fragrance (LF-NPs) were formulated through the self-assembly technique, a simple and low-cost method. The encapsulation of lily fragrance was about 21.9%, and a sustained release was obtained [204]. Another example is the encapsulation of rose fragrance, widely applied in the textile and cosmetics industry, characterised by the presence of many kinds of volatile compounds in this composition. Polybutylcyanoacrylate (PBCA) nanocapsules obtained via anionic polymerisation were successfully used to encapsulate this fragrance (encapsulation efficiency was 65.83%), providing sustained release properties inversely proportional to the nanocapsules size [205]. The same technique has been used for the encapsulation of tuberose fragrance in chitosan nanoparticles characterised by promising controlled release and antibacterial properties [206]. Apple aroma microcapsules were prepared by a complex coacervation–emulsion polymerisation technique using sodium alginate and tetradecylallydimethylammonium bromide as shell materials. The obtained microcapsules have a core-shell structure and a sphere-like shape (diameter from 20 to 50 μm). After the optimisation of the formulation, the microcapsules showed thermal stability up to 110 °C and a 10.8% aroma release after 100 h. The aroma release much increased once the microcapsules were broken by pressure, finding a potential application in cosmetic products [207]. Microcapsules containing camellia oil were prepared using the heterocoagulation approach between chitosan and oleic acid. For the preparation, oleic acid was dissolved in camellia oil and chitosan in the continuous aqueous phase. The obtained core-shell microcapsules were tested as a dressing material to be applied on hair. Their mean diameters ranged from 1.5 μm to 4.5 μm and were adherent on the surface of human hair, being stable both before and after drying [208]. A microparticulate system based on the zein and keratin proteins was developed for the release of fragrances on hair. Linalool and menthol were used as model fragrances. The zein/keratin microparticles were prepared using two approaches: (i) zein nanoparticles were firstly formed, and then, keratin was deposited onto the surface by electrostatic interactions, and (ii) zein was coprecipitated with keratin for microparticle formation. Microparticles were applied onto the hair, forming a film from which fragrances are released, thereby improving the hydration degree and mechanical properties of hair [209]. 

EOs and volatile compounds can be also encapsulated in CDs in order to improve their water solubility; avoid oxygen-, light- or heat-induced degradation and loss during processing and storage and to stabilise them against unwanted changes. Moreover, the use of CD–flavour inclusion complexes allows the use of very small amounts of flavours [210]. 1-Phenylethanol (1-PE) and 1-phenylethanol (2-PE) are important aromatic alcohols with rose-like fragrances that are the major constituents of rose-like flowers scents. The applications of the two isomers have been limited because of their low aqueous solubility, high volatility and thermal instability. For these reasons, CDs have been utilised for the formation of 1:1 stoichiometric inclusion complexes with α-CD, β-CD and HP-β-CD. The results showed that 1-PE and 2-PE can form inclusion complexes with β-CD in a solid state and greatly enhance their stability, indicating that β-CD was a suitable excipient for increasing not only the stability but, also, to achieve a controlled release of 1-PE and 2-PE. Thus, β-CD complexation technology might be a promising approach in terms of expanding the applications of 1-PE and 2-PE [211].

### 3.4. Paper Applications

Another application of the aroma and fragrances encapsulation is the design of aromatic paper or scented paper. Aromatic paper is intended to provide a pleasant surrounding atmosphere on the basis of the aromatherapy principles. In this regard, research has been focused on the development of wallpaper with the aim of providing comfortable sensations and to enhance the psychological and physical well-being [212,213]. Scented papers are, generally, wrapping or writing papers, in which perfumes or fragrances are added for voluptuary purposes or marketing appeal. These papers can be prepared by adding the nano/microspheres containing fragrances or aromas directly into the pulp during the processing operations, or, alternatively, the encapsulated materials can be adsorbed onto the paper surface in a further production step. Moreover, the scented encapsulated compounds can be applied on paper after dispersing them into a coating varnish or ink.

Lavender oil microcapsules were prepared with ABA-type triblock copolymer (polyethylene oxide-polypropylene glycol-polyethylene oxide, PEO-b-PPG-b-PEO) and adsorbed onto the paper surface. The distribution of the microcapsules on the paper surface was homogeneous without degradation. The colour and gloss properties of the paper were also maintained in compliance with the standards [214]. In another work, lavender EO was encapsulated by coacervation using gelatin/gum arabica as the shell material. The obtained microcapsules were dispersed into a UV-curable varnish at a selected microcapsule-to-varnish ratio. The varnish was characterised in terms of the control and protection of the encapsulated lavender EO major volatile components. Notably, the presence of the encapsulated materials does not interfere with the standard screen-printing process generally employed to produce a fragrant gift-wrapping paper [215].

Recently, encapsulated fragrances with an antibacterial effect were applied on paper. Specifically, vanillin was encapsulated in chitosan/poly(lactic-co-glycolic acid (PLGA) nanocapsules to prepare an aromatic wallpaper with an additional antibacterial action. Thanks to the presence of chitosan, the nanospheres showed an antibacterial effect against Gram positive and Gram negative, and the adhesion on the wallpaper was also enhanced [216]. In another work, the encapsulated EO had an antibacterial effect. Citronella EO was encapsulated in microcapsules, obtained by complex coacervation using as a coating material the mixture gelatin/carboxymethyl cellulose or gelatin/gum arabic or by the in-situ polymerisation of melamine–formaldehyde with a polyacrylic acid modifier. These nanocapsules were employed for the preparation of functional coatings intended for paper or cardboard secondary packaging. Both microencapsulation methods provided single-core microcapsules, but some differences were highlighted. Microcapsules from coacervation were more permeable and allowed a steady release of the EO, while those from in-situ polymerisation were impermeable, showing a high retention of the EO, which was released only after a mechanical pressure. The released vapour efficiently inhibited the growth of the tested microorganisms, configuring this manufacture as the first description of a pressure-activated coating for antimicrobial paper [217].

Table 4 summarises the recent studies reporting the microencapsulation and nanoencapsulation of fragrances and flavours for paper applications. 

## 4. Conclusions

Flavours and fragrances are compounds of great importance, widely employed in different products to improve the quality and ameliorate the satisfaction of the consumers. Encapsulation protects them from evaporation and chemical degradation, thereby controlling the release and allowing a simpler handling for processing. This strategy has enabled the use of flavours and fragrances for different technological applications, including the textiles, food, cosmetic and paper industries. Although research is still ongoing in this field, the encapsulation in core-shell polymeric nanoparticles, as well as the formation of molecular inclusion complexes between volatile compounds and CDs, are the most employed techniques in the experimental studies published in the last years. Both techniques resulted in being effective in encapsulating flavours, aroma compounds and EOs in a stable form suitable for different applications. Specifically, remarkable advances have been achieved for the encapsulation of these compounds or their molecular inclusion complexes in micro- or nanofibers/particles via electrodynamic processes. Among all technological fields in which core-shell-encapsulated flavours and fragrances find a relevant usage, the textiles and food packaging industries are the most investigated, despite other applications, such as paper production or coating, that can also benefit from the potential development of these micro- or nanosystems.

## Figures and Tables

**Table 1:** Recent studies reporting the microencapsulation of fragrances and flavours for textile applications. EO: essential oil. CD:cyclodextrin.

Encapsulate System	Carrier Agent	Encapsulated Substance	Encapsulation Method	Applications	Ref.
Microcapsule	Gelatin/Arabic gum	Citronella oil	Complex coacervation	Textiles	[108]
Microcapsule	Gelatin/Arabic gum	Wormwood oil	Complex coacervation	Potential application in health care textiles	[129]
Microcapsule	Chitosan	Citronella oil	Coacervation	Textiles	[114]
Microcapsule	Ethyl cellulose/silica	Lavender fragrance oil	One-step emulsion solvent diffusion	Fragrant and UV resistant textiles	[120]
Microsphere	Cellulose derivatives/Polyvinyl alcohol (PVA)	Eugenol	Oil-in-water emulsion solvent evaporation	Textiles	[130]
Microcapsule	Crosslinked polymers	Model fragrances	Double-emulsion	Potential application in textiles	[27]
Double-Layered Microcapsule	β-CD (inner layer)/chitosan and sodium alginate (outer layer)	Lavender EO	Inclusion encapsulation method	Potential application in textiles	[131]
Microcapsule	Polyurea Shell	Thyme oil	Interfacial polymerisation method	Potential application in textiles	[132]
Microcapsule	Methyltrimethoxysilane/Tetraethyl orthosilicate	Palmarosa oil	Interfacial co-hydrolysis and co-condensation	Health care textiles	[133]
Microcapsule	Melamine	Sage and rose EO	Purchased	Potential application in cosmetotextile	[134]
Microcapsules	Gelatin/Gum acacia	Lemongrass oil	Coacervation	Potential application as antibacterial textile	[135]
Microcapsule	Starch/Glutaraldehyde	*Aloe vera* EO	Coacervation	Functional textiles	[136]
Microcapsule	Gelatin/Gum Arabic	Thyme Oil	Complex coacervation	Potential application as antibacterial textile	[137]
Microcapsule	Silane/orthosilicates/surfactant	Vetiver EO	Interfacial polymerisation technique	Potential application in Health care textiles	[138]
Microcapsule	PVA and Arabic gum and β-CD Inclusion Complex	Tea tree oil	Simple coacervation and inclusion encapsulation	Potential application as antibacterial textiles	[139]
Microcapsule	Acacia gum	Citronella oil	Two-step approach: oil-in-water emulsification and spray-drying	Potential application as cosmetic textiles	[128]
Microcapsule	Polyurethane/β-CD	Neroline	Interfacial polycondensation	Fragrant cosmetotextile	[140]

**Table 2:** Recent studies reporting nanoencapsulation of fragrances and flavours for textile applications.

Encapsulate System	Carrier Agent	Encapsulated Substance	Encapsulation Method	Applications	Ref.
Polymeric micelle	poly(ethylene glycol)-graft poly(vinyl acetate) (PEG-g-PVAc) graft copolymer	2-phenyl ethanol, L-carvone, and α-pinene	Homogenisation	Potential use in textiles	[141]
Nanocapsule	Chitosan	Citronella oil	Coacervation/High-speed homogenisation	Textiles	[114]
Nanoparticle	butyl methacrylate/ethylene glycol dimethacrylate	Limonene	Free-radicalemulsion polymerisation	Potential application in fragrance-release textiles	[29]
Nanocapsule	Epichlorohydrin modified CD	Lavender essence	Purchased	Potential applications as aromatic medical care textiles and household, clothing	[121]
Composite nanoparticle	2-hydroxypropyl-β-CD/regenerated silk fibroin	Limonene Rose Oxide	Complex inclusion/electro-spraying	Fragrant textiles	[113]
Core-shell nanocapsules	Core: styrene/methyl methacrylate copolymer, shell: poly(butyl acrylate) modified by octamethylcyclotetrasiloxane	Jasmine EO	Two-stage emulsion polymerisation	Textile	[142]
Nanofiber	Polyamidoamine dendritic polymer	Thyme EOs	Electrospinning	Potential application as antibacterial textiles	[60]
Nanocapsule	Poly(ε-caprolactone) caprylic/capric triglycerides	Menthol	Nanoprecipitation	Potential application a as cosmetotextiles	[127]

**Table 3:** Recent studies reporting the micro/nanoencapsulation of fragrances and flavours for food applications.

Encapsulate System	Carrier Agent	Encapsulated Substance	Encapsulation Method	Applications	Ref.
Microcapsules	β-CD	Cinnamon and oregano EOs	Solvent evaporation/complex inclusion	Potential application for active packaging	[164]
Nanofibers	Hydroxypropyl-β-CD (HPβCD) and hydroxypropyl-γ-CD (HPγCD)	Cineole and p-cymene	Complex inclusion and electrospinning	Food and oral care applications	[96]
Microcapsule	Different shell materials including jackfruit seed starch, chitosan, and β-CD	Vanilla EO	Ultrasonic method	Potential use in food industry	[165]
Microcapsule	Maltodextrin and gum Arabic	Vanilla and raspberry aromas	Spray-drying	Potential use in food industry	[166]
Inclusion complex	β-CD	Syringa EO	Complex Inclusion	Improving storage of peaches	[151]
Inclusion complex	β-CD	*Eucalyptus staigeriana* Essential Oil	Complex Inclusion	Potential use in food	[167]
Core-shell array	poly(lactic-co-glycolic acid) (PLGA)	Longan milk, or vanilla spices	Ink-Jet Printing	Potential use in food security and anticounterfeiting	[168]
Microcapsule	Gelatin/gum arabic	Pandan flavour	Complex coacervation	Potential use in food industry	[169]
Microsphere	Modified food starch derived from waxy maize	Roasted coffee oil	Spray-drying	Improving quality and acceptance of instant coffee products	[149]
Microcapsule	Chitosan/sodium carboxymethyl cellulose	*Zingiber officinale* EO	Emulsion/freeze-drying	Improving Jujube (*Ziziphus jujuba*) fruit quality	[152]
Microcapsule	Inulin/gum Arabic	*Mentha spicata* EO	Spray-drying	Potential use in pharmaceutical and food applications	[170]
Microcapsule	Carboxymethyl cellulose coating	EOs from *Eucalyptus staigeriana* and *Eucalyptus urograndis*	Mixing	Microorganisms growth control in strawberries	[153]
Microcapsule	Chitosan coating	Savoury and/or tarragon EOs	Mixing	Postharvest maintenance of kumquat (Fortunella sp.) fruit	[171]
Microcapsule	Chitosan coating	*Thymus capitatus* EO	Mixing	Improving shelf-Life of Strawberry during Cold Storage	[172]
Nanocapsule	Chitosan	Pepper tree (*Schinus molle*) EO-loaded	Nanoprecipitation	Postharvest control of *Colletotrichum gloeosporioides* and quality evaluations in avocado	[173]
Inclusion complex	β-CD	Carvacrol, oregano and cinnamon EO	Complex inclusion	Improving the quality of fresh tomatoes during storage through packaging active materials	[174]
Microcapsule	Chitosan coating	Propolis extract and *Zataria multiflora* oil	Mixing	Active packaging of chicken breast meat	[175]
Microparticle	Sodium alginate	Oregano EO	Ionic gelation	Potential use as active and biodegradable packages in food conservation	[176]
Inclusion complex	β-CD	Citral/trans- cinnamaldehyde	Complex inclusion	antimicrobial active packaging for food	[177]
Inclusion complex	β-CD	Watermelon flavour	Complex inclusion	Potential application in food industry	[178]
Microsphere	Chitosan/gum Arabic	Vanilla Oleoresin	Complex coacervation and spray-drying	Potential application in various food matrices	[179]
Nanocapsule	Polybutylcyanoacrylate (PBCA)	Green grass fragrance	Emulsion polymerisation	Potential application in food industry	[180]
Core-shell nanofiber	Zein/tragacanth gum	Saffron extract	Coaxial electrospinning	Potential use in food industry (chewing gum and tea bag development)	[66]
Nanocapsules	Polyurethane	Lavender EO	Emulsion inversion point method	Potential application in food industry	[181]
Nanofiber	chitosan–gelatin	Thyme EO	Nozzleless electrospinning	Nitrite substitute for meat products	[70]
Nanocapsule	Chitosan	*Coriandrum sativum* EO	Emulsion formation/ionic gelation	Prolong shelf life and control the fungal and aflatoxin contamination of stored rice	[182]
Nanoparticle	Polycaprolactone	Geranyl cinnamate	Mini-emulsification/solvent evaporation technique	Potential use antimicrobial packaging	[183]
Nanoparticle	Chitosan	*Paulownia Tomentosa* EO	Ionic gelation method	Improve shelf-life of ready-to-cook pork chops	[184]
Fast-dissolving fibre mats	Balangu seed gum	Bergamot EO	Electrospinning method	Potential strategy for enhancing the flavour in the food systems	[62]
Nanofiber	PVA/β-CD	Cinnamon EO	Electrospinning method	Antimicrobial packaging for fresh mushroom	[160]
Nanosponge	CD	Cinnamon EO	Synthesis via a crosslinking agent	Antimicrobial food packaging	[161]
Nanobiocomposite	Chitosan/β-CD citrate/oxidised nanocellulose	Clove oil	Impregnation of biocomposite	Active food packaging	[162]
Microparticle and nanofiber	Zein	Saffron extract	Electrohydrodynamic processing	Potential use in for active packaging applications or in food formulations.	[163]
Nanofiber in a film	Polylactic acid (PLA)	Thyme EO	Electrospinning	Antimicrobial and humidity sensitive food packaging system	[185]
Nanofiber	Gelatin	Peppermint and chamomile EOs	Electrospinning	Potential application as edible food packaging	[186]
Nanofibrous film	Chitosan/PVA/β-CD	Cinnamon and oregano EOs	Complex inclusion and electrospinning	Potential application in active packaging	[164]
Nanofiber	Zein	Cinnamic aldehyde	Needleless electrospinning	Food additive to reduce nitrites in sausages	[69]

**Table 4:** Recent studies reporting the micro/nanoencapsulation of fragrances and flavours for paper application.

Encapsulate System	Carrier Agent	Encapsulated Substance	Encapsulation Method	Applications	Ref.
Microcapsule	Gelatin/gum Arabic	Lavender EO	Complex coacervation	Making fragrant gift-wrapping paper	[215]
Nanocapsule	Chitosan/PLGA	Vanillin	Emulsion method	Antibacterial function applied to wallpaper	[216]
Nanocapsule	Polyethylene oxide-polypropylene glycol-polyethylene oxide (PEO-b-PPG-b-PEO)	Lavender EO	Micellisation of interactions between the hydrophilic–lipophilic–hydrophilic polymer and oil	Paper coating applications	[214]
Microcapsule	Gelatin/Carboxymethylcellulose or gum arabic	Citronella Oil	Coacervation	Antimicrobial Paper Coatings	[217]
Microcapsule	PLGA or PLGA/Chitosan	Orange EO	Emulsion solvent evaporation	Biodegradable functional packaging paper	[218]

## References

[1] Guentert M. (2007). The flavour and fragrance industry-past, present, and future. Flavours and Fragrances: Chemistry, Bioprocessing and Sustainability.

[2] Bauer K., Garbe D., Surburg H. (1997). Common Fragrance and Flavor Materials: Preparation, Properties and Uses.

[3] Rowe D.J., Rowe D.J. (2004). Chemistry and Technology of Flavors and Fragrances.

[4] Nagegowda D.A., Dudareva N., Kayser O., Quax W.J. (2007). Plant Biochemistry and Biotechnology of Flavor Compounds and Essential Oils. Medicinal Plant Biotechnology: From Basic Research to Industrial Applications.

[5] Can Başer K.H., Buchbauer G. (2015). Handbook of Essential Oils: SCIENCE, Technology, and Applications.

[6] Nieto G. (2017). Biological Activities of Three Essential Oils of the Lamiaceae Family. Medicines.

[7] Herrmann A., Herrmann A. (2010). The Chemistry and Biology of Volatiles.

[8] Weerawatanakorn M., Wu J.C., Pan M.H., Ho C.T. (2015). Reactivity and stability of selected flavor compounds. J. Food Drug Anal..

[9] Branta D., Speranza L.P., Macedo G.A. (2016). A new approach for flavor and aroma encapsulation. Novel Approaches of Nanotechnology in Food Nanotechnology in the Agri-Food Industry Volume 1 Nanotechnology in the Agri-Food Industry.

[10] Van Soest J.J.G. (2007). Encapsulation of fragrances and flavours: A way to control odour and aroma in consumer products. Flavours and Fragrances: Chemistry, Bioprocessing and Sustainability.

[11] Estevinho B.N., Roch F., Oprea A.E., Grumezescu A.M. (2017). A Key for the Future of the Flavors in Food Industry: Nanoencapsulation and Microencapsulation. Nanotechnology Applications in Food Flavor, Stability, Nutrition and Safety.

[12] Chakraborty S. (2017). Carrageenan for encapsulation and immobilization of flavor, fragrance, probiotics, and enzymes: A review. J. Carbohydr. Chem..

[13] Ciriminna R., Pagliaro M. (2013). Sol-gel microencapsulation of odorants and flavors: Opening the route to sustainable fragrances and aromas. Chem. Soc. Rev..

[14] Sadovoy A.V., Lomova M.V., Antipina M.N., Braun N.A., Sukhorukov G.B., Kiryukhin M.V. (2013). Layer-by-layer assembled multilayer shells for encapsulation and release of fragrance. ACS Appl. Mater. Interfaces.

[15] Kaur R., Kukkar D., Bhardwaj S.K., Kim K.H., Deep A. (2018). Potential use of polymers and their complexes as media for storage and delivery of fragrances. J. Control. Release.

[16] He L., Hu J., Deng W. (2018). Preparation and application of flavor and fragrance capsules. Polym. Chem..

[17] Dardelle G., Jacquemond M., Erni P. (2017). Delivery Systems for Low Molecular Weight Payloads: Core/Shell Capsules with Composite Coacervate/Polyurea Membranes. Adv. Mater..

[18] Yow H.N., Routh A.F. (2006). Formation of liquid core-polymer shell microcapsules. Soft Matter.

[19] Esser-Kahn A.P., Odom S.A., Sottos N.R., White S.R., Moore J.S. (2011). Triggered release from polymer capsules. Macromolecules.

[20] Paret N., Trachsel A., Berthier D.L., Herrmann A. (2019). Developing Multi Stimuli-Responsive Core/Shell Microcapsules to Control the Release of Volatile Compounds. Macromol. Mater. Eng..

[21] Lee H., Choi C.-H., Abbaspourrad A., Wesner C., Caggioni M., Zhu T., Weitz D.A. (2016). Encapsulation and Enhanced Retention of Fragrance in Polymer Microcapsules. ACS Appl. Mater. Interfaces.

[22] Ciobanu A., Landy D., Fourmentin S. (2013). Complexation efficiency of cyclodextrins for volatile flavor compounds. Food Res. Int..

[23] Cabral-Marques H. (2010). A review on cyclodextrin encapsulation of essential oils and volatiles. Flavour Fragr. J..

[24] Wadhwa G., Kumar S., Chhabra L., Mahant S., Rao R. (2017). Essential oil–cyclodextrin complexes: An updated review. J. Incl. Phenom. Macrocycl. Chem..

[25] Saifullah, Shishir M.R.I., Ferdowsi R., Rahman R.T., Vuong Q. (2019). Micro and nano encapsulation, retention and controlled release of flavor and aroma compounds: A critical review. Trends Food Sci. Technol..

[26] Wei M., Pan X., Rong L., Dong A., He Y., Song X., Li J. (2020). Polymer carriers for controlled fragrance release. Mater. Res. Express.

[27] Stasse M., Ribaut T., Héroguez V., Schmitt V. (2020). Elaboration of double emulsion-based polymeric capsules for fragrance. Colloid Polym. Sci..

[28] Ji Z., Tang Y., Wang C., Yang J. (2020). Preparation of submicron capsules containing fragrance and their application as emulsifier. Polym. Bull..

[29] Manfredini N., Ilare J., Invernizzi M., Polvara E., Mejia D.C., Sironi S., Moscatelli D., Sponchioni M. (2020). Polymer Nanoparticles for the Release of Fragrances: How the Physicochemical Properties Influence the Adsorption on Textile and the Delivery of Limonene. Ind. Eng. Chem. Res..

[30] Stasse M., Laurichesse E., Vandroux M., Ribaut T., Héroguez V., Schmitt V. (2020). Cross-linking of double oil-in-water-in-oil emulsions: A new way for fragrance encapsulation with tunable sustained release. Colloids Surf. A Physicochem. Eng. Asp..

[31] Timilsena Y.P., Akanbi T.O., Khalid N., Adhikari B., Barrow C.J. (2019). Complex coacervation: Principles, mechanisms and applications in microencapsulation. Int. J. Biol. Macromol..

[32] Aloys H., Korma S.A., Alice Tuyishime M., Ali A.H., Marie Alice T., Chantal N., Abed S.M., Ildephonse H. (2016). Microencapsulation by Complex Coacervation: Methods, Techniques, Benefits, and Applications-A Review. Am. J. Food Sci. Nutr. Res..

[33] Martins I.M., Barreiro M.F., Coelho M.A., Rodrigues A.E. (2014). Microencapsulation of essential oils with biodegradable polymeric carriers for cosmetic applications. Chem. Eng. J..

[34] Bakry A.M., Abbas S., Ali B., Majeed H., Abouelwafa M.Y., Mousa A., Liang L. (2015). Microencapsulation of Oils: A Comprehensive Review of Benefits, Techniques, and Applications. Compr. Rev. Food Sci. Food Saf..

[35] Niu Y., Liu W., Zhu G., Zhou R., Niu Y. (2013). A review of the preparation and application of flavour and essential oils microcapsules based on complex coacervation technology. J. Sci. Food Agric..

[36] Muhoza B., Xia S., Wang X., Zhang X., Li Y., Zhang S. (2020). Microencapsulation of essential oils by complex coacervation method: Preparation, thermal stability, release properties and applications. Crit. Rev. Food Sci. Nutr..

[37] Zhang Y., Ma J., Xu Q. (2019). Polyelectrolyte complex from cationized casein and sodium alginate for fragrance controlled release. Colloids Surf. B Biointerfaces.

[38] Hernández-Nava R., López-Malo A., Palou E., Ramírez-Corona N., Jiménez-Munguía M.T. (2020). Encapsulation of oregano essential oil (Origanum vulgare) by complex coacervation between gelatin and chia mucilage and its properties after spray drying. Food Hydrocoll..

[39] Ammendola M., Gomez R.R., Garcia-Valls R. (2019). Perfume Encapsulation via Vapor Induced Phase Separation. Processes.

[40] Gupta S., Khan S., Muzafar M., Kushwaha M., Yadav A.K., Gupta A.P. (2016). Encapsulation: Entrapping essential oil/flavors/aromas in food. Encapsulations.

[41] Anandharamakrishnan C., Ishwarya S.P. (2015). Spray Drying Techniques for Food Ingredient Encapsulation.

[42] Estevinho B.N., Rocha F., Santos L., Alves A. (2013). Microencapsulation with chitosan by spray drying for industry applications–A review. Trends Food Sci. Technol..

[43] Reineccius G.A., Yan C. (2015). Factors controlling the deterioration of spray dried flavourings and unsaturated lipids. Flavour Fragr. J..

[44] Veiga R.D.S.D., Da Silva-Buzanello R.A., Corso M.P., Canan C. (2019). Essential oils microencapsulated obtained by spray drying: A review. J. Essent. Oil Res..

[45] Mohammed N.K., Tan C.P., Manap M.Y.A., Muhialdin B.J., Hussin A.S.M. (2020). Spray Drying for the Encapsulation of Oils—A Review. Molecules.

[46] Assadpour E., Jafari S.M. (2019). Advances in Spray-Drying Encapsulation of Food Bioactive Ingredients: From Microcapsules to Nanocapsules. Annu. Rev. Food Sci. Technol..

[47] Hu Q., Li X., Chen F., Wan R., Yu C., Li J., McClements D.J., Deng Z.-Y. (2020). Microencapsulation of an essential oil (cinnamon oil) by spray drying: Effects of wall materials and storage conditions on microcapsule properties. J. Food Process. Preserv..

[48] Ramos F.D.M., Júnior V.S., Prata A.S. (2019). Assessing the Vacuum Spray Drying Effects on the Properties of Orange Essential Oil Microparticles. Food Bioprocess Technol..

[49] Procopio F.R., Oriani V., Paulino B.N., Prado-Silva L.D., Pastore G.M., Sant’Ana A.S., Hubinger M.D. (2018). Solid lipid microparticles loaded with cinnamon oleoresin: Characterization, stability and antimicrobial activity. Food Res. Int..

[50] Sillick M., Gregson C.M. (2012). Spray chill encapsulation of flavors within anhydrous erythritol crystals. LWT.

[51] Đorđević V., Balanc B.D., Belščak-Cvitanović A., Lević S., Trifković K., Kalušević A., Kostić I., Komes D., Bugarski B., Nedović V. (2015). Trends in Encapsulation Technologies for Delivery of Food Bioactive Compounds. Food Eng. Rev..

[52] Coelho S.C., Estevinho B.N., Rocha F. (2020). Encapsulation in food industry with emerging electrohydrodynamic techniques: Electrospinning and electrospraying–A review. Food Chem..

[53] Haider S., Haider A., Alghyamah A.A., Khan R., Almasry W.A., Khan N. (2019). Electrohydrodynamic Processes and Their Affecting Parameters. Electrospinning and Electrospraying-Techniques and Applications.

[54] Wen P., Wen Y., Zong M.-H., Linhardt R.J., Wu H. (2017). Encapsulation of Bioactive Compound in Electrospun Fibers and Its Potential Application. J. Agric. Food Chem..

[55] Ataei S., Azari P., Hassan A., Pingguan-Murphy B., Yahya R., Muhamad F. (2020). Essential Oils-Loaded Electrospun Biopolymers: A Future Perspective for Active Food Packaging. Adv. Polym. Technol..

[56] Solaberrieta I., Jiménez A., Cacciotti I., Garrigós M. (2020). Encapsulation of Bioactive Compounds from Aloe Vera Agrowastes in Electrospun Poly (Ethylene Oxide) Nanofibers. Polymer.

[57] Mele E. (2020). Electrospinning of Essential Oils. Polymer.

[58] Ullah A., Saito Y., Ullah S., Haider K., Nawaz H., Duy-Nam P., Kharaghani D., Kim I.-S. (2020). Bioactive Sambong oil-loaded electrospun cellulose acetate nanofibers: Preparation, characterization, and in-vitro biocompatibility. Int. J. Biol. Macromol..

[59] Venturelli R., Immich A.P., De Souza S., De Souza A.A. (2020). Recycled polyester nanofiber as a reservoir for essential oil release. J. Appl. Polym. Sci..

[60] Kamrudi N., Akbari S., Kish M.H. (2020). Enhanced control release of thyme essential oils from electrospun nanofiber/polyamidoamine dendritic polymer for antibacterial platforms. Polym. Adv. Technol..

[61] Dehghani S., Noshad M., Rastegarzadeh S., Hojjati M., Fazlara A. (2020). Electrospun chia seed mucilage/PVA encapsulated with green cardamonmum essential oils: Antioxidant and antibacterial property. Int. J. Biol. Macromol..

[62] Rezaeinia H., Emadzadeh B., Ghorani B. (2020). Electrospun balangu (Lallemantia royleana) hydrocolloid nanofiber mat as a fast-dissolving carrier for bergamot essential oil. Food Hydrocoll..

[63] Buzgo M., Mickova A., Rampichova M., Doupnik M. (2018). Blend electrospinning, coaxial electrospinning, and emulsion electrospinning techniques. Core-Shell Nanostructures for Drug Delivery and Theranostics.

[64] Yao Z.-C., Chen S., Ahmad Z., Huang J., Chang M.-W., Li J. (2017). Essential Oil Bioactive Fibrous Membranes Prepared via Coaxial Electrospinning. J. Food Sci..

[65] Camerlo A., Vebert-Nardin C., Rossi R.M., Popa A.-M. (2013). Fragrance encapsulation in polymeric matrices by emulsion electrospinning. Eur. Polym. J..

[66] Dehcheshmeh M.A., Fathi M. (2019). Production of core-shell nanofibers from zein and tragacanth for encapsulation of saffron extract. Int. J. Biol. Macromol..

[67] Jung Y., Yang H., Lee I.Y., Yong T.-S., Lee S. (2020). Core/Sheath-Structured Composite Nanofibers Containing Cinnamon Oil: Their Antibacterial and Antifungal Properties and Acaricidal Effect against House Dust Mites. Polymer.

[68] Yu M., Dong R.-H., Yan X., Yu G.-F., You M.-H., Ning X., Long Y.-Z. (2017). Recent Advances in Needleless Electrospinning of Ultrathin Fibers: From Academia to Industrial Production. Macromol. Mater. Eng..

[69] Karim M., Fathi M., Soleimanian-Zad S. (2020). Nanoencapsulation of cinnamic aldehyde using zein nanofibers by novel needle-less electrospinning: Production, characterization and their application to reduce nitrite in sausages. J. Food Eng..

[70] Vafania B., Fathi M., Soleimanian-Zad S. (2019). Nanoencapsulation of thyme essential oil in chitosan-gelatin nanofibers by nozzle-less electrospinning and their application to reduce nitrite in sausages. Food Bioprod. Process..

[71] Castro N., Durrieu V., Raynaud C., Rouilly A., Rigal L., Quellet C. (2016). Melt Extrusion Encapsulation of Flavors: A Review. Polym. Rev..

[72] Hoyos-Leyva J.D., Bello-Pérez L.A., Alvarez-Ramirez J., Garcia H.S. (2018). Microencapsulation using starch as wall material: A review. Food Rev. Int..

[73] Zhang J., Normand V. (2020). Gelatinization of octenyl succinate starch affects oil encapsulation in melt extrusion. LWT.

[74] Fahim T., Zaidul I., Abu Bakar M.R., Salim U., Awang M., Sahena F., Jalal K., Sharif K., Sohrab M. (2014). Particle formation and micronization using non-conventional techniques- review. Chem. Eng. Process. Process. Intensif..

[75] Soh S.H., Lee L.Y. (2019). Microencapsulation and Nanoencapsulation Using Supercritical Fluid (SCF) Techniques. Pharmaceutics.

[76] Akolade J., Nasir-Naeem K.O., Swanepoel A., Yusuf A.A., Balogun M., Labuschagne P. (2020). CO2-assisted production of polyethylene glycol/lauric acid microparticles for extended release of Citrus aurantifolia essential oil. J. CO2 Util..

[77] Akolade J., Balogun M., Swanepoel A., Ibrahim R.B., A Yusuf A., Labuschagne P. (2019). Microencapsulation of eucalyptol in polyethylene glycol and polycaprolactone using particles from gas-saturated solutions. RSC Adv..

[78] Reis P.M.C.L., Mezzomo N., Aguiar G.P.S., Senna E.M.T.L., Hense H., Ferreira S.R.S. (2019). Ultrasound-assisted emulsion of laurel leaves essential oil (Laurus nobilis L.) encapsulated by SFEE. J. Supercrit. Fluids.

[79] Tian B., Xiao D., Hei T., Ping R., Hua S., Liu J. (2020). The application and prospects of cyclodextrin inclusion complexes and polymers in the food industry: A review. Polym. Int..

[80] Matencio A., Navarro-Orcajada S., García-Carmona F., López-Nicolás J.M. (2020). Applications of cyclodextrins in food science. A review. Trends Food Sci. Technol..

[81] Haji A. (2020). Functional Finishing of Textiles with β-Cyclodextrin. Frontiers of Textile Materials.

[82] Bezerra F.M., Lis M., Firmino H.B., Da Silva J.G.D., Valle R.D.C.S.C., Valle J.A.B., Scacchetti F.A.P., Tessaro A.L. (2020). The Role of β-Cyclodextrin in the Textile Industry—Review. Molecules.

[83] Behr A., Seidensticker T. (2020). Cyclic Carbohydrates-Cyclodextrins. Chemistry of Renewables.

[84] Rodríguez-López M.I., Mercader-Ros M.T., Lucas-Abellán C., Pellicer J.A., Pérez-Garrido A., Pérez-Sánchez H., Yáñez-Gascón M.J., Gabaldón J., Núñez-Delicado E. (2020). Comprehensive Characterization of Linalool-HP-β-Cyclodextrin Inclusion Complexes. Molecules.

[85] Niu Y., Deng J., Xiao Z., Kou X., Zhu G., Liu M., Liu S. (2020). Preparation and slow release kinetics of apple fragrance/β-cyclodextrin inclusion complex. J. Therm. Anal. Calorim..

[86] Zhu G., Zhu G., Xiao Z. (2020). Study of formation constant, thermodynamics and β-ionone release characteristic of β-ionone-hydroxypropyl-β-cyclodextrin inclusion complex. Polym. Bull..

[87] Christoforides E., Fourtaka K., Andreou A., Bethanis K. (2020). X-ray crystallography and molecular dynamics studies of the inclusion complexes of geraniol in β-cyclodextrin, heptakis (2,6-di-O-methyl)-β-cyclodextrin and heptakis (2,3,6-tri-O-methyl)-β-cyclodextrin. J. Mol. Struct..

[88] Kfoury M., Auezova L., Greige-Gerges H., Fourmentin S. (2019). Encapsulation in cyclodextrins to widen the applications of essential oils. Environ. Chem. Lett..

[89] Saffarionpour S. (2019). Nanoencapsulation of Hydrophobic Food Flavor Ingredients and Their Cyclodextrin Inclusion Complexes. Food Bioprocess Technol..

[90] Yildiz Z.I., Celebioglu A., Kilic M.E., Durgun E., Uyar T. (2018). Fast-dissolving carvacrol/cyclodextrin inclusion complex electrospun fibers with enhanced thermal stability, water solubility, and antioxidant activity. J. Mater. Sci..

[91] Celebioglu A., Yildiz Z.I., Uyar T. (2018). Thymol/cyclodextrin inclusion complex nanofibrous webs: Enhanced water solubility, high thermal stability and antioxidant property of thymol. Food Res. Int..

[92] Celebioglu A., Aytac Z., Kilic M.E., Durgun E., Uyar T. (2017). Encapsulation of camphor in cyclodextrin inclusion complex nanofibers via polymer-free electrospinning: Enhanced water solubility, high temperature stability, and slow release of camphor. J. Mater. Sci..

[93] Yildiz Z.I., Celebioglu A., Kilic M.E., Durgun E., Uyar T. (2018). Menthol/cyclodextrin inclusion complex nanofibers: Enhanced water-solubility and high-temperature stability of menthol. J. Food Eng..

[94] Aytac Z., Yildiz Z.I., Kayaci-Senirmak F., Keskin N.O.S., Kusku S.I., Durgun E., Tekinay T., Uyar T. (2016). Fast-Dissolving, Prolonged Release, and Antibacterial Cyclodextrin/Limonene-Inclusion Complex Nanofibrous Webs via Polymer-Free Electrospinning. J. Agric. Food Chem..

[95] Aytac Z., Celebioglu A., Yildiz Z.I., Uyar T. (2018). Efficient Encapsulation of Citral in Fast-Dissolving Polymer-Free Electrospun Nanofibers of Cyclodextrin Inclusion Complexes: High Thermal Stability, Longer Shelf-Life, and Enhanced Water Solubility of Citral. Nanomaterials.

[96] Celebioglu A., Yildiz Z.I., Uyar T. (2017). Electrospun nanofibers from cyclodextrin inclusion complexes with cineole and p -cymene: Enhanced water solubility and thermal stability. Int. J. Food Sci. Technol..

[97] Celebioglu A., Yildiz Z.I., Uyar T. (2018). Fabrication of Electrospun Eugenol/Cyclodextrin Inclusion Complex Nanofibrous Webs for Enhanced Antioxidant Property, Water Solubility, and High Temperature Stability. J. Agric. Food Chem..

[98] Aytac Z., Ipek S., Durgun E., Tekinay T., Uyar T. (2017). Antibacterial electrospun zein nanofibrous web encapsulating thymol/cyclodextrin-inclusion complex for food packaging. Food Chem..

[99] Wang Z., Zou W., Liu L., Wang M., Li F., Shen W. (2020). Characterization and bacteriostatic effects of β-cyclodextrin/quercetin inclusion compound nanofilms prepared by electrospinning. Food Chem..

[100] Celebioglu A., Uyar T. (2021). Electrohydrodynamic encapsulation of eugenol-cyclodextrin complexes in pullulan nanofibers. Food Hydrocoll..

[101] Chen Y., Mensah A., Wang Q., Li D., Qiu Y., Weic Q. (2020). Hierarchical porous nanofibers containing thymol/beta-cyclodextrin: Physico-chemical characterization and potential biomedical applications. Mater. Sci. Eng. C.

[102] Figueroa-Lopez K.J., Enescu D., Torres-Giner S., Cabedo L., Cerqueira M., Pastrana L., Fuciños P., Lagaron J.M. (2020). Development of electrospun active films of poly(3-hydroxybutyrate-co-3-hydroxyvalerate) by the incorporation of cyclodextrin inclusion complexes containing oregano essential oil. Food Hydrocoll..

[103] Ghayempour S., Montazer M. (2016). Micro/nanoencapsulation of essential oils and fragrances: Focus on perfumed, antimicrobial, mosquito-repellent and medical textiles. J. Microencapsul..

[104] Teixeira C.S.N.R., Martins I.M.D., Mata V.L.G., Barreiro M.F.F., Rodrigues A.E. (2011). Characterization and evaluation of commercial fragrance microcapsules for textile application. J. Text. Inst..

[105] Teixeira M.A., Rodríguez O., Rodrigues S., Martins I., Rodrigues A.E. (2011). A case study of product engineering: Performance of microencapsulated perfumes on textile applications. AIChE J..

[106] Wang C.X., Chen S.L. (2005). Fragrance-release Property of β-Cyclodextrin Inclusion Compounds and their Application in Aromatherapy. J. Ind. Text..

[107] Schindler W.D., Hauser P.J. (2004). Chemical Finishing of Textiles.

[108] Bezerra F.M., Lis M., Óscar C.G., Carmona C.G., Moisés M.P., Zanin G.M., Moraes F.F. (2019). Assessment of the delivery of citronella oil from microcapsules supported on wool fabrics. Powder Technol..

[109] Zhao D., Jiao X., Zhang M., Ye K., Shi X., Lu X., Qiu G., Shea K.J. (2016). Preparation of high encapsulation efficiency fragrance microcapsules and their application in textiles. RSC Adv..

[110] Bruyninckx K., Dusselier M. (2019). Sustainable Chemistry Considerations for the Encapsulation of Volatile Compounds in Laundry-Type Applications. ACS Sustain. Chem. Eng..

[111] Cerempei A. (2017). Aromatherapeutic Textiles. Active Ingredients from Aromatic and Medicinal Plants.

[112] Rodrigues S., Martins I.M., Fernandes I.P., Gomes P., Mata V., Barreiro M.F., Rodrigues A.E. (2009). Scentfashion^®^: Microencapsulated perfumes for textile application. Chem. Eng. J..

[113] Ye L., Li Z., Niu R., Zhou Z., Shen Y., Jiang L. (2019). All-Aqueous Direct Deposition of Fragrance-Loaded Nanoparticles onto Fabric Surfaces by Electrospraying. ACS Appl. Polym. Mater..

[114] Liu X., Huang L., Chen H., Qian M.C., Ji H. (2019). Pore size matching up: A novel insight into cotton textile aromatic finishing. Flavour Fragr. J..

[115] Romagnoli M.J., Gonzalez J.S., Martinez M.A., Alvarez V. (2020). Micro- and Nanotechnology Applied on Eco-friendly Smart Textiles. Handbook of Nanomaterials and Nanocomposites for Energy and Environmental Applications.

[116] Sharkawy A., Fernandes I.P., Barreiro M.F., Rodrigues A.E., Shoeib T. (2017). Aroma-Loaded Microcapsules with Antibacterial Activity for Eco-Friendly Textile Application: Synthesis, Characterization, Release, and Green Grafting. Ind. Eng. Chem. Res..

[117] Ghayempour S., Mortazavi S.M. (2015). Microwave curing for applying polymeric nanocapsules containing essential oils on cotton fabric to produce antimicrobial and fragrant textiles. Cellulose.

[118] Specos M.M., García J., Tornesello J., Marino P., Della Vecchia M., Tesoriero M.D., Hermida L.G. (2010). Microencapsulated citronella oil for mosquito repellent finishing of cotton textiles. Trans. R. Soc. Trop. Med. Hyg..

[119] Eyupoglu S., Kut D., Girisgin A.O., Eyupoglu C., Ozuicli M., Dayioglu H., Civan M., Aydin L. (2019). Investigation of the bee-repellent properties of cotton fabrics treated with microencapsulated essential oils. Text. Res. J..

[120] Chen K., Xu C., Zhou J., Zhao R., Gao Q., Wang C. (2020). Multifunctional fabric coatings with slow-releasing fragrance and UV resistant properties from ethyl cellulose/silica hybrid microcapsules. Carbohydr. Polym..

[121] Ma J., Xu W., Kou X., Niu Y., Xia Y., Wang Y., Tian G., Zhao Y., Ke Q. (2020). Green fabrication of control-released, washable and non-adhesives aromatic-nanocapsules/cotton fabrics via electrostatic-adsorption/in-situ immobilization. ACS Sustain. Chem. Eng..

[122] Persico P., Carfagna C. (2012). Cosmeto-Textiles: State of the Art and Future Perspectives. Adv. Sci. Technol..

[123] Mamta M., Saini H.K., Kaur M. (2017). Cosmetotextiles: A novel technique of developing wearable skin care. Asian J. HOME Sci..

[124] Cheng S., Yuen C., Kan C., Cheuk K. (2008). Development of Cosmetic Textiles Using Microencapsulation Technology. Res. J. Text. Appar..

[125] Jamal Z., Rani S., Zeba C., Hd J.P., Scholars R. (2018). Cosmetotextiles: A wearable skin care. Int. J. Home Sci..

[126] Gonçalves F., Ribeiro A., Silva C., Cavaco-Paulo A. (2019). Release of Fragrances from Cotton Functionalized with Carbohydrate-Binding Module Proteins. ACS Appl. Mater. Interfaces.

[127] Yingngam B., Chiangsom A., Pharikarn P., Vonganakasame K., Kanoknitthiran V., Rungseevijitprapa W., Prasitpuriprecha C. (2019). Optimization of menthol-loaded nanocapsules for skin application using the response surface methodology. J. Drug Deliv. Sci. Technol..

[128] Yingngam B., Kacha W., Rungseevijitprapa W., Sudta P., Prasitpuriprecha C., Brantner A.H. (2019). Response surface optimization of spray-dried citronella oil microcapsules with reduced volatility and irritation for cosmetic textile uses. Powder Technol..

[129] Wang L., Li T., Xin B., Liu Y., Zhang F. (2020). Preparation and characterization of wormwood-oil-contained microcapsules. J. Microencapsul..

[130] Simões M., Coimbra P., Carreira A.S., Figueiredo M.M., Gil M.H., Simões P.N. (2020). Eugenol-loaded microspheres incorporated into textile substrates. Cellulose.

[131] Zhang T., Luo Y., Wang M., Chen F., Liu J., Meng K., Zhao H. (2020). Double-Layered Microcapsules Significantly Improve the Long-Term Effectiveness of Essential Oil. Polymer.

[132] Wang X., Li C., Wang M., Zhao T., Li W. (2020). Bifunctional Microcapsules with n-Octadecane/Thyme Oil Core and Polyurea Shell for High-Efficiency Thermal Energy Storage and Antibiosis. Polymer.

[133] Kudligi S.J., Malligawad L.H., Naikwadi S., Jamadar D. (2020). Antimicrobial and aroma finishing of organic cotton knits using natural colourants and Palmarosa oil microcapsules. Flavour Fragr. J..

[134] Stan M.S., Chirila L., Popescu A., Radulescu D.M., Radulescu D.E., Dinischiotu A. (2019). Essential Oil Microcapsules Immobilized on Textiles and Certain Induced Effects. Materials.

[135] Bhatt L., Singh S.S.J. (2018). Comparative analysis of lemongrass oil application on textile substrate through microencapsulation and exhaust method. Int. J. Adv. Res. Sci. Eng..

[136] Fiedler J.O., Óscar C.G., Carmona C.G., Lis M.J., Plath A.M.S., Samulewski R.B., Bezerra F.M. (2020). Application of Aloe vera microcapsules in cotton nonwovens to obtain biofunctional textiles. J. Text. Inst..

[137] Karagönlü S., Başal G., Özyıldız F., Uzel A. (2018). Preparation of Thyme Oil Loaded Microcapsules for Textile Applications. Int. J. New Technol. Res..

[138] Sannapapamma K.J., Lokanath H.M., Naikwadi S. (2018). Antimicrobial and Aroma Finishing of Organic Cotton Knits Using Vetiver Oil Microcapsules for Health Care Textiles. Int. J. Mater. Text. Eng..

[139] Beşen B.S. (2019). Production of Disposable Antibacterial Textiles Via Application of Tea Tree Oil Encapsulated into Different Wall Materials. Fibers Polym..

[140] Ben Abdelkader M., Azizi N., Baffoun A., Chevalier Y., Majdoub M. (2019). Fragrant Microcapsules Based On β-Cyclodextrin for Cosmetotextile Application. J. Renew. Mater..

[141] Mamusa M., Sofroniou C., Resta C., Murgia S., Fratini E., Smets J., Baglioni P. (2020). Tuning the Encapsulation of Simple Fragrances with an Amphiphilic Graft Copolymer. ACS Appl. Mater. Interfaces.

[142] Liu C., Liang B., Wang Y., Li Y., Shi G. (2018). Core-shell nanocapsules containing essential oil for textile application. J. Appl. Polym. Sci..

[143] Đorđević V., Paraskevopoulou A., Mantzouridou F., Lalou S., Pantic M., Bugarski B., Nedović V. (2015). Encapsulation Technologies for Food Industry. Food Engineering Series.

[144] Kayaci F., Uyar T. (2012). Encapsulation of vanillin/cyclodextrin inclusion complex in electrospun polyvinyl alcohol (PVA) nanowebs: Prolonged shelf-life and high temperature stability of vanillin. Food Chem..

[145] Maswal M., Dar A.A. (2014). Formulation challenges in encapsulation and delivery of citral for improved food quality. Food Hydrocoll..

[146] Singh T., Shukla S., Kumar P., Wahla V., Bajpai V.K., Rather I.A. (2017). Application of Nanotechnology in Food Science: Perception and Overview. Front. Microbiol..

[147] Sanguansri L., Augustin M.A. (2010). Microencapsulation in Functional Food Product Development. Functional Food Product Development.

[148] Rajabi H., Ghorbani M., Jafari S.M., Mahoonak A.S., Rajabzadeh G. (2015). Retention of saffron bioactive components by spray drying encapsulation using maltodextrin, gum Arabic and gelatin as wall materials. Food Hydrocoll..

[149] Zanin R.C., Smrke S., Kurozawa L.E., Yamashita F., Yeretzian C. (2020). Modulation of aroma release of instant coffees through microparticles of roasted coffee oil. Food Chem..

[150] Froiio F., Mosaddik A., Morshed M.T., Paolino D., Fessi H., Elaissari A. (2019). Edible Polymers for Essential Oils Encapsulation: Application in Food Preservation. Ind. Eng. Chem. Res..

[151] Yang W., Wang L., Ban Z., Yan J., Lu H., Zhang X., Wu Q., Aghdam M.S., Luo Z., Li L. (2019). Efficient microencapsulation of Syringa essential oil; the valuable potential on quality maintenance and storage behavior of peach. Food Hydrocoll..

[152] Ban Z., Zhang J., Li L., Luo Z., Wang Y., Yuan Q., Zhou B., Liu H. (2020). Ginger essential oil-based microencapsulation as an efficient delivery system for the improvement of Jujube (Ziziphus jujuba Mill.) fruit quality. Food Chem..

[153] Da Silva P.P.M., De Oliveira J., Biazotto A.D.M., Parisi M.M., Da Gloria E.M., Spoto M.H.F. (2020). Essential oils from Eucalyptus staigeriana F. Muell. ex Bailey and Eucalyptus urograndis W. Hill ex Maiden associated to carboxymethylcellulose coating for the control of Botrytis cinerea Pers. Fr. and Rhizopus stolonifer (Ehrenb.:Fr.) Vuill. in strawberries. Ind. Crop. Prod..

[154] Yildirim S., Röcker B., Pettersen M.K., Nilsen-Nygaard J., Ayhan Z., Rutkaite R., Radusin T., Suminska P., Marcos B., Coma V. (2018). Active Packaging Applications for Food. Compr. Rev. Food Sci. Food Saf..

[155] Wyrwa J., Barska A. (2017). Innovations in the food packaging market: Active packaging. Eur. Food Res. Technol..

[156] De Abreu D.A.P., Cruz J.M., Losada P.P. (2012). Active and Intelligent Packaging for the Food Industry. Food Rev. Int..

[157] Cruz-Romero M., Murphy T.V., Morris M.J., Cummins E., Kerry J.P. (2013). Antimicrobial activity of chitosan, organic acids and nano-sized solubilisates for potential use in smart antimicrobially-active packaging for potential food applications. Food Control..

[158] Sharma S., Barkauskaite S., Jaiswal A.K., Jaiswal S. (2020). Essential oils as additives in active food packaging. Food Chem..

[159] Zhao L., Duan G., Zhang G., Yang H., He S., Jiang S. (2020). Electrospun Functional Materials toward Food Packaging Applications: A Review. Nanomaterials.

[160] Pan J., Ai F., Shao P., Chen H., Gao H. (2019). Development of polyvinyl alcohol/β-cyclodextrin antimicrobial nanofibers for fresh mushroom packaging. Food Chem..

[161] Simionato I., Domingues F., Nerin C., Silva F. (2019). Encapsulation of cinnamon oil in cyclodextrin nanosponges and their potential use for antimicrobial food packaging. Food Chem. Toxicol..

[162] Adel A.M., Ibrahim A.A., El-Shafei A.M., Al-Shemy M. (2019). Inclusion complex of clove oil with chitosan/β-cyclodextrin citrate/oxidized nanocellulose biocomposite for active food packaging. Food Packag. Shelf Life.

[163] Alehosseini A., Gómez-Mascaraque L.G., Ghorani B., Lopez-Rubio A. (2019). Stabilization of a saffron extract through its encapsulation within electrospun/electrosprayed zein structures. LWT.

[164] Munhuweyi K., Caleb O.J., Van Reenen A.J., Opara U.L. (2018). Physical and antifungal properties of β-cyclodextrin microcapsules and nanofibre films containing cinnamon and oregano essential oils. LWT.

[165] Zhu H., Zhang Y., Tian J., Chu Z. (2018). Effect of a new shell material—Jackfruit seed starch on novel flavor microcapsules containing vanilla oil. Ind. Crop. Prod..

[166] Jedlińska A., Samborska K., Janiszewska-Turak E., Witrowa-Rajchert D., Seuvre A.-M., Voilley A. (2018). Physicochemical properties of vanilla and raspberry aromas microencapsulated in the industrial conditions by spray drying. J. Food Process. Eng..

[167] Yuan C., Thomas D.S., Hook J.M., Qin G., Qi K., Zhao J. (2019). Molecular Encapsulation of Eucalyptus staigeriana Essential Oil by Forming Inclusion Complexes with Hydroxypropyl-β-Cyclodextrin. Food Bioprocess Technol..

[168] Chen B., Su M., Pan Q., Zhang Z., Chen S., Huang Z., Cai Z., Li Z., Qian X., Hu X. (2019). Fully Printed Geranium-Inspired Encapsulated Arrays for Quantitative Odor Releasing. ACS Omega.

[169] Samakradhamrongthai R.S., Angeli P.T., Kopermsub P., Utama-Ang N. (2019). Optimization of gelatin and gum arabic capsule infused with pandan flavor for multi-core flavor powder encapsulation. Carbohydr. Polym..

[170] Mehran M., Masoum S., Memarzadeh M. (2020). Microencapsulation of Mentha spicata essential oil by spray drying: Optimization, characterization, release kinetics of essential oil from microcapsules in food models. Ind. Crop. Prod..

[171] Hosseini S.F., Amraie M., Salehi M., Mohseni M., Aloui H. (2019). Effect of chitosan-based coatings enriched with savory and/or tarragon essential oils on postharvest maintenance of kumquat (Fortunella sp.) fruit. Food Sci. Nutr..

[172] Martínez K., Ortiz M., Albis A., Castañeda C.G.G., Zapata M.E.V., Tovar C.D.G. (2018). The Effect of Edible Chitosan Coatings Incorporated with Thymus capitatus Essential Oil on the Shelf-Life of Strawberry (Fragaria x ananassa) during Cold Storage. Biomol..

[173] Chávez-Magdaleno M.E., González-Estrada R., Ramos-Guerrero A., Plascencia-Jatomea M., Gutierrez-Martinez P. (2018). Effect of pepper tree (Schinus molle) essential oil-loaded chitosan bio-nanocomposites on postharvest control of Colletotrichum gloeosporioides and quality evaluations in avocado (Persea americana) cv. Hass. Food Sci. Biotechnol..

[174] Buendía-Moreno L., Ros-Chumillas M., Navarro-Segura L., Sánchez-Martínez M.J., Soto-Jover S., Antolinos V., Martínez-Hernández G.B., López–Gómez A. (2019). Effects of an Active Cardboard Box Using Encapsulated Essential Oils on the Tomato Shelf Life. Food Bioprocess Technol..

[175] Mehdizadeh T., Langroodi A.M. (2019). Chitosan coatings incorporated with propolis extract and Zataria multiflora Boiss oil for active packaging of chicken breast meat. Int. J. Biol. Macromol..

[176] Paglione I.D.S., Galindo M.V., De Medeiros J.A.S., Yamashita F., Alvim I.D., Grosso C.R.F., Sakanaka L.S., Shirai M.A. (2019). Comparative study of the properties of soy protein concentrate films containing free and encapsulated oregano essential oil. Food Packag. Shelf Life.

[177] Chen H., Li L., Ma Y., McDonald T.P., Wang Y. (2019). Development of active packaging film containing bioactive components encapsulated in β-cyclodextrin and its application. Food Hydrocoll..

[178] Xiao Z., Hou W., Kang Y., Niu Y., Kou X. (2019). Encapsulation and sustained release properties of watermelon flavor and its characteristic aroma compounds from γ-cyclodextrin inclusion complexes. Food Hydrocoll..

[179] Hernández-Fernández M.Á., García-Pinilla S., Ocampo-Salinas O.I., Gutiérrez-López G.F., Hernández-Sánchez H., Cornejo-Mazón M., Perea-Flores M.D.J., Dávila-Ortiz G. (2020). Microencapsulation of Vanilla Oleoresin (*V. planifolia* Andrews) by Complex Coacervation and Spray Drying: Physicochemical and Microstructural Characterization. Foods.

[180] Zhu G.Y., Lin C.T., Chen J.M., Lei D.M. (2018). The study of size and stability of n-butylcyanoacrylate nanocapsule suspensions encapsulating green grass fragrance. IOP Conf. Series: Mater. Sci. Eng..

[181] Cui G., Wang J., Wang X., Li W., Zhang X. (2018). Preparation and Properties of Narrowly Dispersed Polyurethane Nanocapsules Containing Essential Oil via Phase Inversion Emulsification. J. Agric. Food Chem..

[182] Das S., Singh V.K., Dwivedy A.K., Chaudhari A.K., Upadhyay N., Singh P., Sharma S., Dubey N.K. (2019). Encapsulation in chitosan-based nanomatrix as an efficient green technology to boost the antimicrobial, antioxidant and in situ efficacy of Coriandrum sativum essential oil. Int. J. Biol. Macromol..

[183] Zanetti M., Mazon L.R., De Meneses A.C., Silva L.L., De Araújo P.H.H., Fiori M.A., De Oliveira D. (2019). Encapsulation of geranyl cinnamate in polycaprolactone nanoparticles. Mater. Sci. Eng. C.

[184] Zhang H., Li X., Kang H. (2019). Chitosan coatings incorporated with free or nano-encapsulated Paulownia Tomentosa essential oil to improve shelf-life of ready-to-cook pork chops. LWT.

[185] Min T., Sun X., Yuan Z., Zhou L., Jiao X., Zha J., Zhu Z., Wen Y. (2021). Novel antimicrobial packaging film based on porous poly(lactic acid) nanofiber and polymeric coating for humidity-controlled release of thyme essential oil. LWT.

[186] Xu Y., Zhou Y., Lan X., Huang D., Luo T., Ji J., Mafang Z., Miao X., Wang H., Wang W. (2019). Electrospun Gelatin Nanofibers Encapsulated with Peppermint and Chamomile Essential Oils as Potential Edible Packaging. J. Agric. Food Chem..

[187] European Parliament & Council of the European Union (2009). Regulation (EC) NO 1223/2009 of the European Parliament and of the Council on Cosmetic Products. Off. J. Eur. Union.

[188] Global Beauty & Personal Care Market | Trends, Growth, Size, Share. https://www.inkwoodresearch.com/reports/beauty-and-personal-care-market/.

[189] Beauty Becomes a $532 billion Industry Thanks to These trends-Business Insider. https://www.businessinsider.com/beauty-multibillion-industry-trends-future-2019-7?IR=T.

[190] Carvalho I.T., Estevinho B.N., Santos L.R.D.A.D. (2016). Application of microencapsulated essential oils in cosmetic and personal healthcare products - a review. Int. J. Cosmet. Sci..

[191] Corrêa-Filho L.C., Moldão-Martins M., Alves Vitor D. (2019). Advances in the Application of Microcapsules as Carriers of Functional Compounds for Food Products. Appl. Sci..

[192] Casanova F., Santos L. (2016). Encapsulation of cosmetic active ingredients for topical application—a review. J. Microencapsul..

[193] Gonçalves G.M.S., Srebernich S.M., Vercelino B.G., Zampieri B.M. (2013). Influence of the presence and type of fragrance on the sensory perception of cosmetic formulations. Braz. Arch. Biol. Technol..

[194] Tumová J., Šauer P., Golovko O., Koba Ucun O., Grabic R., Máchová J., Kocour Kroupová H. (2019). Effect of polycyclic musk compounds on aquatic organisms: A critical literature review supplemented by own data. Sci. Total Environ..

[195] Ammala A. (2012). Biodegradable polymers as encapsulation materials for cosmetics and personal care markets. Int. J. Cosmet. Sci..

[196] Aranaz I., Acosta N., Civera C., Elorza B., Mingo J., Castro C., Civera C., Heras A. (2018). Cosmetics and Cosmeceutical Applications of Chitin, Chitosan and Their Derivatives. Polym..

[197] Mohsenabadi N., Rajaei A., Tabatabaei M., Mohsenifar A. (2018). Physical and antimicrobial properties of starch-carboxy methyl cellulose film containing rosemary essential oils encapsulated in chitosan nanogel. Int. J. Biol. Macromol..

[198] Devi N., Sarmah M., Khatun B., Maji T.K. (2017). Encapsulation of active ingredients in polysaccharide–protein complex coacervates. Adv. Colloid Interface Sci..

[199] Van Tran V., Nguyen T.L., Moon J.-Y., Lee Y.-C. (2019). Core-shell materials, lipid particles and nanoemulsions, for delivery of active anti-oxidants in cosmetics applications: Challenges and development strategies. Chem. Eng. J..

[200] Kesente M., Kavetsou E., Roussaki M., Blidi S., Loupassaki S., Chanioti S., Siamandoura P., Stamatogianni C., Philippou E., Papaspyrides C. (2017). Encapsulation of Olive Leaves Extracts in Biodegradable PLA Nanoparticles for Use in Cosmetic Formulation. Bioengineering.

[201] Dos Santos C., Álvaro V., Fronza N., Dos Santos J.H.Z. (2017). Structural, textural and morphological characteristics of tannins from Acacia mearnsii encapsulated using sol-gel methods: Applications as antimicrobial agents. Colloids Surf. B Biointerfaces.

[202] Sansukcharearnpon A., Wanichwecharungruang S., Leepipatpaiboon N., Kerdcharoen T., Arayachukeat S. (2010). High loading fragrance encapsulation based on a polymer-blend: Preparation and release behavior. Int. J. Pharm..

[203] Triyono K., Suhartatik N., Wulandari Y.W. (2018). Nanoencapsulating of Kaffir Lime Oil with Coacervation Method using Arabic Gum and Maltodextrin as Encapsulant. Int. J. Food Nutr. Sci..

[204] Ji W., Zhang T., Lu Z., Shen J., Xiao Z., Zhang X. (2019). Synthesis and characterization of novel biocompatible nanocapsules encapsulated lily fragrance. Chin. Chem. Lett..

[205] Hu J., Xiao Z., Zhou R., Li Z., Wang M., Ma S. (2011). Synthesis and characterization of polybutylcyanoacrylate-encapsulated rose fragrance nanocapsule. Flavour Fragr. J..

[206] Xiao Z., Tian T., Hu J., Wang M., Zhou R. (2013). Preparation and characterization of chitosan nanoparticles as the delivery system for tuberose fragrance. Flavour Fragr. J..

[207] Song J., Chen H. (2018). Preparation of aroma microcapsules with sodium alginate and tetradecylallyldimethylammonium bromide (TADAB) and its potential applications in cosmetics. Flavour Fragr. J..

[208] Saito N., Taguchi Y., Tanaka M. (2018). Preparation of Microcapsules Containing Camellia Oil with Heterocoagulation between Chitosan and Oleic Acid. J. Cosmet. Dermatol. Sci. Appl..

[209] Tinoco A., Gonçalves F., Costa A.F., Freitas D.S., Cavaco-Paulo A., Ribeiro A. (2020). Keratin:Zein particles as vehicles for fragrance release on hair. Ind. Crop. Prod..

[210] Yang S., Liu L., Han J., Tang Y. (2020). Encapsulating plant ingredients for dermocosmetic application: An updated review of delivery systems and characterization techniques. Int. J. Cosmet. Sci..

[211] Yang Z., Yao X., Xiao Z., Chen H., Ji H. (2015). Preparation and release behaviour of the inclusion complexes of phenylethanol withβ-cyclodextrin. Flavour Fragr. J..

[212] Niu Y., Zhang Y., Zhu G., Zhou R., Niu Y. (2017). Preparation and sustained-releasing characterization of aromatic wallpaper. Prog. Org. Coat..

[213] Xiao Z., Zhang Y., Zhu G., Niu Y., Xu Z., Zhu J. (2017). Preparation of micro-encapsulated strawberry fragrance and its application in the aromatic wallpaper. Pol. J. Chem. Technol..

[214] Kandirmaz E.A., Birtane H., Çiğil A.B., Ozcan A. (2020). pH-controlled lavender oil capsulation with ABA-type block copolymer and usage in paper coating. Flavour Fragr. J..

[215] Rungwasantisuk A., Raibhu S. (2020). Application of encapsulating lavender essential oil in gelatin/gum-arabic complex coacervate and varnish screen-printing in making fragrant gift-wrapping paper. Prog. Org. Coat..

[216] Zhang T., Lu Z., Yang J., Wang J., Shen J., Wang X., Xiao Z., Niu Y., Chen L., Zhang X. (2020). Chitosan-based nanofragrance with antibacterial function applied to wallpaper. Eng. Life Sci..

[217] Šumiga, Ravnjak D., Bojana B.P. (2019). Antimicrobial Paper Coatings Containing Microencapsulated Cymbopogon citratus Oil. Coatings.

[218] Xiao Z., Wan S., Niu Y., Kou X. (2019). Effect of Preparation Parameters on Microparticles with High Loading Capacity and Adsorption Property Adsorbed on Functional Paper. Coatings.

